# QResearch primary care records linked to national hospital and cancer registry data: a validation study

**DOI:** 10.1016/j.esmorw.2026.100763

**Published:** 2026-08-24

**Authors:** S. Kulkarni, V. Perletta, Z. Wang, J.W. Tomlinson, J. Hippisley-Cox, G.S. Collins, A.K. Clift, D. Dodwell, S.R. Lord

**Affiliations:** 1 Department of Oncology, University of Oxford, Oxford, UK; 2 Nuffield Department of Population Health, University of Oxford, Oxford, UK; 3 Radcliffe Department of Medicine, Oxford Centre for Diabetes, Endocrinology and Metabolism, NIHR Oxford Biomedical Research Centre, University of Oxford, Oxford, UK; 4 Wolfson Institute of Population Health, Queen Mary University of London, London, UK; 5 Department of Applied Health Sciences, School of Health Sciences, College of Medicine and Health, University of Birmingham, Birmingham, UK; 6 NIHR Birmingham Biomedical Research Centre, University Hospitals Birmingham NHS Foundation Trust and University of Birmingham, Birmingham, UK; 7 Department of Surgery and Cancer, Imperial College London, London, UK

**Keywords:** cancer registry, data linkage study, electronic health records, primary care, real-world data, systemic anticancer therapy

## Abstract

**Background:**

Population-based linkage of primary care electronic health records to oncology-relevant datasets enables detailed analysis of cancer care and outcomes. QResearch is a large primary care database containing longitudinal health records from a subset of general practices in England, linked to national hospital, cancer registry, treatment and mortality data. However, the extent to which this primary care-hospital-registry linkage accurately reflects the national cancer population and cancer treatment patterns has not been validated.

**Materials and methods:**

This validation study included adults aged ≥18 years registered at QResearch-contributing practices with an incident diagnosis of breast, prostate, colorectal, melanoma, kidney, pancreatic or gastro-oesophageal cancer between 2013 and 2020. Demographic characteristics and proportions treated with systemic anticancer therapy (SACT) or radiotherapy (RT) were compared with national statistics. We evaluated, in the absence of the cancer registry, the ascertainment and representativeness of patients with cancer identifiable in other sources.

**Results:**

Between 2013 and 2020, the QResearch-linked dataset captured 14.6% (*n* = 196 565/1 348 243) of national cancer registrations across seven cancer sites. Distributions of sex, age and stage closely matched national data, with regional variation reflecting participating practices. Proportions treated with SACT and RT aligned with national estimates, with differences typically within 2%. The 93.8% of cases identifiable without registry data remained demographically and clinically representative, with minimal impact on overall cohort characteristics.

**Conclusion:**

The QResearch-linked dataset offers a nationally representative and comprehensive platform for population oncology research. Its integration of detailed primary care information, including comorbidities, medications and lifestyle factors, should enable evaluation of treatment predictors and outcomes across diverse populations.

## Highlights

• QResearch primary care data were linked to hospital, registry and mortality data.
• The dataset captured 14.6% of national registrations across seven cancer types.
• Age, sex and stage distributions closely matched national cancer statistics.
• Systemic therapy and RT proportions aligned with national statistics.
• The linked dataset is nationally representative for oncology research.

## Introduction

Large-scale linkage of routinely collected health data has transformed population oncology research, enabling detailed study of cancer incidence, treatment patterns and outcomes at scale, including the development of risk prediction models.^1^^,^^2^ Linked datasets combining primary care, hospital and registry data offer opportunities to characterise comorbidity, medication exposure and lifestyle factors that would not be possible using administrative registries in isolation.^3^^,^^4^ Primary care electronic health records (EHRs) provide information on multimorbidity and lifestyle factors that are unavailable in administrative registries and essential for real-world cancer research.^4^^,^^5^ In particular, England is well placed for such integration with some of the most comprehensive population-based health care and cancer-specific datasets available internationally.^6^, ^7^, ^8^, ^9^

### National cancer datasets in England

In England, the National Disease Registration Service (NDRS) collects near-complete population-based data on almost all National Health Service (NHS) patients with malignant tumours through the National Cancer Registration Dataset (NCRD or ‘cancer registry’).^6^ With national coverage since 1971, the NCRD incorporates diagnostic, staging and treatment information, including for systemic anticancer therapy (SACT) and radiotherapy (RT).^6^ Data are derived from multiple secondary care feeds such as hospital episode statistics (HES),^7^ the SACT dataset,^8^ and the national radiotherapy dataset (RTDS).^6^^,^^10^^,^^11^ Early SACT dataset submissions were affected by variable completeness and interhospital variation, highlighting the importance of multisource linkage.^12^^,^^13^

Annual cancer registration statistics in England are routinely published and provide quality-assured estimates of incidence by cancer site, demographics and geography.^6^^,^^14^ Given their completeness, these statistics constitute the benchmark against which to assess the representativeness of linked EHR cancer cohorts in England.

### QResearch-linked cancer dataset

QResearch is one of the largest primary care EHR databases worldwide, comprising pseudonymised data from over 1500 general practices in England and covering >40 million ever-registered patients.^9^^,^^15^ In 2018, QResearch included 13.1 million currently registered patients, equivalent to approximately one-fifth of the current population of England. Participating practices are geographically distributed across England, although practice participation and coverage vary by region. The database includes detailed patient-level information on demographics, diagnoses, comorbidities, prescribed medications, laboratory results and lifestyle factors, with longitudinal follow-up extending from 1990.^15^ QResearch has also been individually linked to external sources of centrally collected national data, including HES admitted patient care (APC) and Outpatient data, the NCRD, the SACT dataset and national death registrations. Linkage is carried out deterministically using pseudonymised NHS numbers, which have been reported to be over 99.8% complete within QResearch records.^16^

QResearch-linked data have already underpinned several cancer risk prediction and prognostic modelling studies within oncology, such as those estimating breast cancer-specific mortality.^10^^,^^17^, ^18^, ^19^, ^20^, ^21^, ^22^, ^23^, ^24^ However, despite its growing use, validation of linked QResearch-cancer data against national cancer statistics has not previously been published.

### Need for validation

EHRs are primarily collected for delivery of direct clinical care rather than for research purposes. Secondary data use therefore poses several well-recognised challenges related to representativeness, completeness and data quality.^5^^,^^25^ Potential biases may arise from variation in practice recruitment, coding practices and data completeness. Furthermore, cancer registry releases are subject to time lags, as they are highly curated.^6^ Evaluating the representativeness of cohorts identified using alternative linked sources is therefore essential for ensuring the validity of longitudinal population-based studies.

In the QResearch-linked cancer dataset, the underlying population is defined by patients registered with participating general practices, with the potential for some geographic and demographic variation compared with the national population. At the time of analysis, linkage to the NCRD was available on 31 December 2020, whereas QResearch, HES, SACT and death registration data extended beyond this date. Understanding the characteristics and treatment patterns of patients with cancer identifiable within the linked dataset without the NCRD is essential for interpreting studies conducted when registry data are not available. We therefore used the fully linked 2013-2020 period to evaluate, in the absence of the cancer registry, the ascertainment and representativeness of cancer cases recorded in hospital and primary care records.

### Study aims

1. Determine whether the QResearch-linked cancer dataset is demographically representative of national cancer registration data for seven common cancer sites.
2. Determine whether the proportion of patients with cancer receiving systemic therapy and RT in the linked dataset is nationally representative against NDRS statistics.
3. Evaluate, in the absence of the NCRD, the ascertainment and representativeness of cancer cases recorded in hospital, primary care and death registration records.

## Materials and methods

### Study design

We conducted a retrospective, population-based validation study using primary care data from QResearch linked to secondary care and cancer registry data. Analyses evaluated the representativeness of patient demographics and proportions treated with SACT and RT compared with NDRS cancer statistics, and whether excluding tumours identified only in the NCRD affected cohort composition and representativeness.

### Data sources

**Figure 1:**
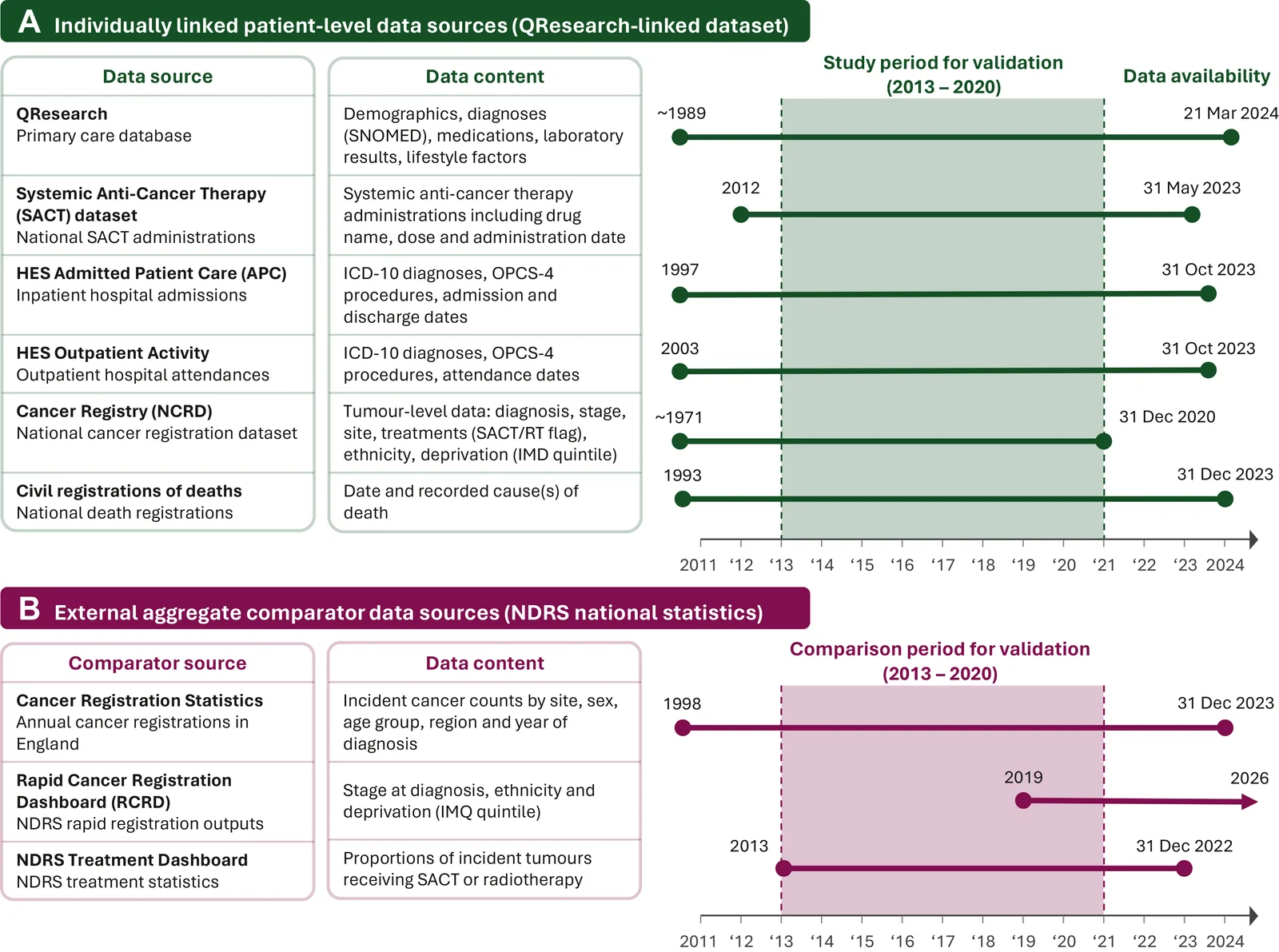
**Overview of linked patient-level and external aggregate comparator data sources used for validation of the QResearch-linked cancer dataset.** (A) The national datasets linked at individual patient-level within the QResearch-linked cancer dataset, including QResearch primary care records, HES APC and Outpatient datasets, the NCRD, the SACT dataset, and civil registrations of deaths. Horizontal bars indicate the period of data availability for each source, with the shaded area denoting the study validation period (1 January 2013 to 31 December 2020) when all linked and comparator datasets were available. (B) The external aggregate comparator datasets obtained from the NDRS and used to assess representativeness of the linked cohort. Cancer Registration Statistics in England were used to compare distributions of incident cancers by site, age, sex, region and year of diagnosis (2013-2020); the RCRD was used to compare stage at diagnosis, ethnicity and deprivation distributions (2019-2020); and the NDRS Treatment Dashboard was used to compare proportions of tumours receiving SACT and RT (2013-2020). APC, admitted patient care; HES, hospital episode statistics; IMD, index of multiple deprivation; NCRD, National Cancer Registration Dataset; NDRS, National Disease Registration Service; QResearch, QResearch primary care database; RCRD, Rapid Cancer Registration Dashboard; RT, radiotherapy; SACT, systemic anticancer therapy.

Data were linked at an individual level using five national data sources in England that have been used extensively in cancer epidemiology,^6^, ^7^, ^8^, ^9^^,^^26^^,^^27^ as outlined in Figure 1:

• **QResearch**: a large pseudonymised database of general practice records derived from practices using the Egton Medical Information System in England. It includes demographics, comorbidities, diagnoses, prescriptions, laboratory results and lifestyle factors, with patients followed until death, deregistration from their contributing practice or the last available QResearch extract (21 March 2024).
• **HES APC and outpatient**: records of inpatient and outpatient attendances at NHS hospitals in England, including Office of Population Censuses and Surveys Classification of Interventions and Procedures (OPCS-4) procedures, International Classification of Diseases (ICD)-10 diagnoses and ethnicity.
• **NCRD**: tumour-level records for malignant neoplasms in England, with national coverage since 1971 and near-complete ascertainment of incident cancer diagnoses.^6^ Extracted variables included tumour site and stage at diagnosis, ethnicity, area-level deprivation [index of multiple deprivation (IMD)] and, treatment flags denoting receipt of SACT or RT within defined attribution periods.^28^
• **SACT dataset**: national systemic therapy activity reported by NHS trusts in England, with initial submissions from 2012 and a near-complete national coverage achieved by 2014.^12^
• **Civil registrations of deaths**: national death registration data for England.

These linked datasets were compared against published national cancer statistics from the NDRS, as outlined in Figure 1. These publicly available summary statistics are derived from England’s population-based cancer registration system, which integrates multiple national data feeds and quality assurance processes and has an estimated case ascertainment of 98%-99%.^6^^,^^29^ The following benchmark outputs were used:

• **Cancer Registration Statistics (England, 2013-2020)**, providing incident counts by cancer site, age group, sex, region and year of diagnosis.^6^
• **The Rapid Cancer Registration Dashboard (2019-2020)**, used to obtain comparator data for ethnicity, stage and deprivation distributions for the included cancer sites; denominators may differ from final statistics due to rapid reporting.^30^
• **The NDRS treatment dashboard (England, 2013-2020)**, which reports proportions of incident tumours receiving SACT or RT, defined using NDRS attribution rules.^10^

### Study population

The study population comprised adults aged ≥18 years registered with a QResearch-contributing general practice between 1 January 2013 and 31 December 2023, with at least 1 year continuous registration. Patients were included if they had a cancer record in any of QResearch, HES, NCRD or the death register. Patients were followed from diagnosis until the end of the tumour-specific timeframe (defined below) or death. Analyses were restricted to tumours diagnosed between 1 January 2013 and 31 December 2020, when linked data from all sources were available.

To enable comparisons between the QResearch-linked cancer data and NDRS national statistics, methods used in calculating national cancer statistics were followed as strictly as possible. Analyses were limited to seven cancer sites with matching ICD-10 definitions between NDRS and QResearch code groups: breast (C50), prostate (C61), colorectal (C18-C20), melanoma (C43), kidney (C64-C66, C68), pancreas (C25) and gastro-oesophageal (C15-C16). Male patients with gynaecological cancers, female patients with prostate cancers, and those with benign tumours were excluded. Cancer sites for which exact alignment of ICD-10 definitions between the predefined QResearch code groups available within the approved data extract and NDRS comparator outputs was not possible, including lung cancer, were excluded because heterogeneity in case definitions would be a source of bias likely affecting interpretation.

Cancer diagnoses in QResearch were identified using Systematized Nomenclature of Medicine - Clinical Terms (SNOMED-CT) code groups mapped to ICD-10 site definitions, whereas HES and death registration diagnoses used ICD-10 codes. HES procedures, including administration of SACT and RT, were identified using OPCS-4 codes,^7^ whereas SACT treatment records were defined using the national SACT dataset standard, including dm+d medicine coding.^8^^,^^31^ Tumour-level variables such as stage were obtained from the NCRD where available, as these are not consistently captured in non-registry sources. Although stage is recorded for many patients within the SACT dataset, it is available only for patients receiving systemic therapy, reflects stage at treatment initiation rather than diagnosis, and has recognised data quality limitations.^8^

Further exclusions aligned with NDRS definitions and comparators. For demographic comparisons, cases recorded only in QResearch (without a linked NCRD, HES or death record) were excluded. For treatment comparisons, patients with insufficient follow-up to cover the modality-specific attribution windows were additionally excluded (see below). As HES and SACT data are patient-level, tumours in patients with another primary cancer within 18 months before or after diagnosis were excluded, in line with NDRS methodology.^28^

### Identification of treatments

We applied NDRS-defined treatment attribution windows from 1 month before to 18 months after diagnosis, depending on site and modality (Supplementary Table S1, https://doi.org/10.1016/j.esmorw.2026.100763). Undated registry treatment flags contradicted by dated records from other sources were removed. Treatments were identified in line with NDRS methodology as follows^28^:

• SACT was identified from the SACT dataset (excluding supportive therapies such as bisphosphonates and oral hormone therapies), HES OPCS chemotherapy codes and the chemotherapy flag within the NCRD.
• RT was identified using HES OPCS RT codes and the RT flag in the NCRD.

### Data analysis

This study is reported in accordance with the STrengthening the Reporting of OBservational studies in Epidemiology (STROBE) guidelines. Analyses were conducted in Stata 18 (StataCorp LLC, College Station, TX). Categorical variables were summarised as counts and percentages; 95% confidence intervals (CIs) were calculated using exact binomial methods, with nonoverlapping CIs interpreted as statistically significant.

Demographic representativeness was assessed by comparing distributions of sex, age group, year of diagnosis, region, stage, ethnicity and deprivation (IMD quintile) between the QResearch-linked dataset and NDRS national statistics. Ethnicity, stage and deprivation comparisons were restricted to diagnoses in 2019-2020.

For treatment comparisons, we calculated site- and year-specific proportions receiving SACT or RT within attribution windows and compared these with corresponding NDRS statistics. Given the known incompleteness in SACT dataset submissions before 2015,^12^^,^^13^ we disaggregated the relative contributions of SACT, HES and NCRD treatment flags to overall treatment capture.

We identified cancers ascertainable through QResearch, HES or death registrations and characterised ‘NCRD-only’ tumours, defined as cases recorded in the NCRD but not in QResearch, HES or death registrations. Given tumour-level recording in NCRD but patient-level recording in other sources, we anticipated that some NCRD-only cases could represent subsequent tumours of the same site that cannot be reliably identified elsewhere. Therefore, we conducted a first-cancer sensitivity analysis to reestimate the proportion and characteristics of NCRD-only tumours.

## Results

### Cohort derivation

**Figure 2:**
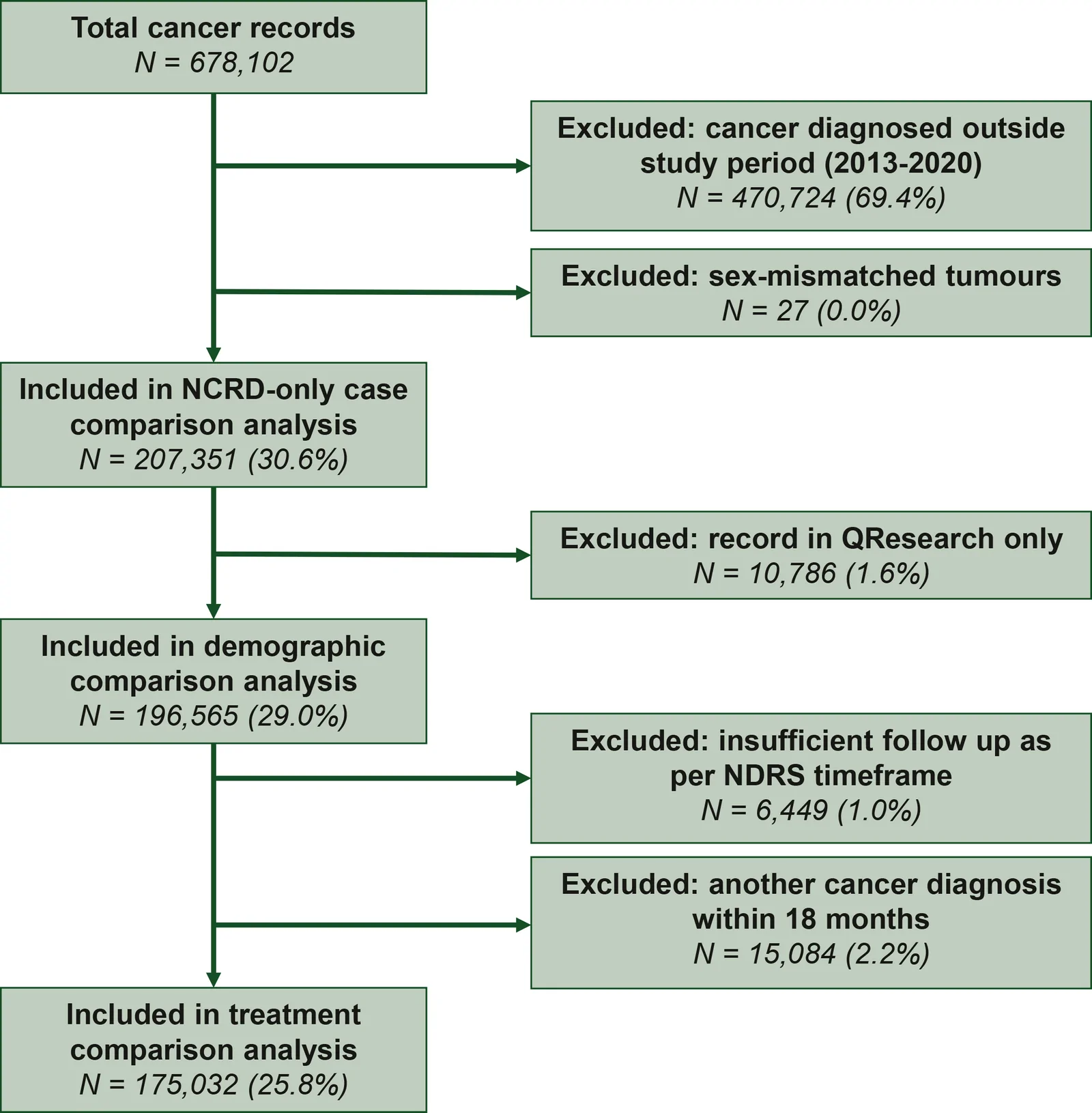
**Cohort derivation.** Flowchart shows sequential exclusions and resulting cohort size at each stage. Total cancers refer to all primary malignant tumours identified in QResearch, HES, the NCRD or death records. Final analytic cohorts differ by analysis time to align with exclusions applied by national comparator datasets. HES, hospital episode statistics; NCRD, National Cancer Registration Dataset; NDRS, National Disease Registration Service; QResearch, QResearch primary care database.

The derivation of the analytic cohorts is shown in Figure 2. In total, 678 102 cancer records were identified for the seven cancer sites across all linked sources. After restricting to the study period and excluding sex mismatches, 207 351 cases were eligible for evaluating cancer ascertainment without the NCRD, 196 565 cases contributed to demographic comparisons, and 175 032 records were included in analyses of treated proportions.

### Demographic representativeness

The demographic characteristics of cases in the QResearch-linked dataset and corresponding national statistics are summarised in Table 1, with stratification by cancer site presented in Supplementary Table S2 (https://doi.org/10.1016/j.esmorw.2026.100763). Overall, the linked dataset captured 14.6% (*n* = 196 565/1 348 243) of all eligible cancers in England across the seven tumour sites during 2013-2020. Captured proportions increased from 11.9% in 2013 to 17.2% in 2020, consistent with expansion of QResearch practice participation.

**Table 1:** Demographic characteristics of patients in the QResearch-linked dataset compared with NDRS national cancer registration statistics in England, calendar years 2013-2020

Demographics	QResearch, *N* (%)	NDRS, *N* (%)
Total cancers (row %)	196 565 (14.6)	1 348 243
Cancer type (row %)		
Breast	50 832 (13.9)	366 009
Prostate	47 790 (14.2)	335 439
Colorectal	40 818 (14.6)	280 321
Melanoma	16 831 (15.5)	108 646
Kidney	13 793 (16.1)	85 884
Pancreatic	11 077 (16.0)	69 086
Gastro-oesophageal	15 424 (15.0)	102 858
Sex		
Male	104 253 (53.0)	707 447 (52.5)
Female	92 313 (47.0)	640 796 (47.5)
Age at diagnosis (years)		
<50	17 348 (8.8)	126 978 (9.4)
50-59	28 838 (14.7)	199 955 (14.8)
60-69	49 410 (25.1)	346 442 (25.7)
70-79	58 101 (29.6)	387 983 (28.8)
≥80	42 868 (21.8)	286 885 (21.3)
Year of diagnosis (row %)		
2013	19 271 (11.9)	161 891
2014	20 499 (12.5)	164 429
2015	22 616 (13.6)	166 436
2016	23 820 (14.2)	167 169
2017	25 674 (15.3)	168 175
2018	28 102 (15.5)	181 463
2019	29 619 (16.2)	183 298
2020	26 694 (17.2)	155 382
Region of residence		
East Midlands	3792 (1.9)	118 406 (8.8)
East of England	8882 (4.5)	159 802 (11.9)
London	32 640 (16.6)	147 843 (11.0)
North East	5586 (2.8)	68 969 (5.1)
North West	43 068 (21.9)	178 760 (13.3)
South East	52 957 (26.9)	240 421 (17.8)
South West	20 711 (10.5)	160 631 (11.9)
West Midlands	23 031 (11.7)	141 849 (10.5)
Yorkshire and Humber	5898 (3.0)	131 562 (9.8)
NB. Below data only available for 2019-2020 (inclusive)	56 583	Ethnicity: 315 461 Stage: 327 994 IMD: 338 680
Ethnicity		
White	48 626 (91.6)	282 208 (92.6)
Asian	1727 (3.3)	8715 (2.9)
Black	1311 (2.5)	7295 (2.4)
Other	1412 (2.7)	6379 (2.1)
Unknown	3507	10 864
Stage		
Unstageable	49 (0.1)	421 (0.2)
Stages 1 and 2	24 982 (58.9)	159 505 (59.5)
Stages 3 and 4	17 405 (41.0)	108 032 (40.3)
Unknown	12 867	60 036
IMD quintile		
1 (most deprived)	7967 (15.0)	51 136 (15.1)
2	9878 (18.6)	60 687 (17.9)
3	10 821 (20.3)	71 250 (21.0)
4	11 500 (21.6)	76 625 (22.6)
5 (least deprived)	13 059 (24.5)	78 982 (23.3)
Unknown	3358	0

Values represent counts (%) unless otherwise indicated. Percentages for known groups are shown to enable valid comparison of distributions with national statistics. Column percentages reported unless otherwise specified. Ethnicity data derived from the Rapid Cancer Registration Dashboard (2019-2020); numbers therefore differ from NDRS totals. Stage data unavailable for some ICD-10 code subsets in kidney and for gastric cancers and therefore population sizes may differ.

ICD, International Classification of Diseases; IMD, index of multiple deprivation; NDRS, National Disease Registration Service; QResearch, QResearch primary care database.

Distributions of sex and age group at diagnosis were closely aligned to national statistics (53.0% versus 52.5% male, 8.8% versus 9.4% <50 years, 14.7% versus 14.8% 50-59 years, 25.1% versus 25.7% 60-69 years, 29.6% versus 28.8% 70-79 years, and 21.8% versus 21.3% 80+ years in QResearch-linked data versus NDRS, respectively). For the 2019-2020 subset, stage, ethnicity and deprivation also showed close concordance.

Regional patterns reflected the geographic distribution of participating QResearch practices,^15^ with higher case capture in London, the North West and the South East. Nonetheless, all nine English regions were represented and regional clustering did not materially impact the age, sex, ethnicity or deprivation profile of the cohort.

### Comparison of treated proportions

#### SACT

**Figure 3:**
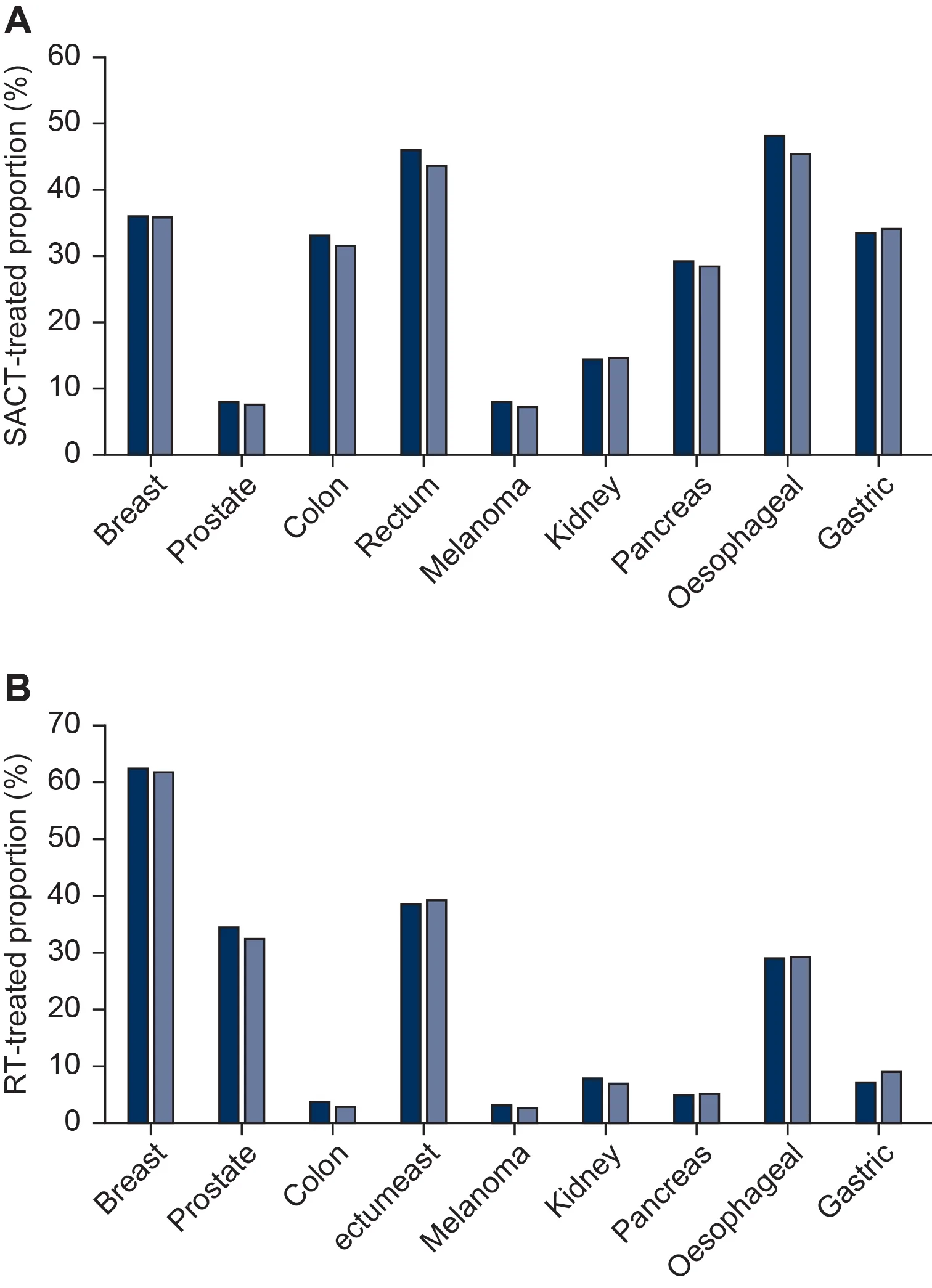
**Clustered bar chart comparing treated proportions between the QResearch-linked dataset and NDRS national statistics, calendar years 2013-2020.** (A) SACT, (B) RT. NDRS, National Disease Registration Service; QResearch, QResearch primary care database; RT, radiotherapy; SACT, systemic anticancer therapy.

**Figure 4:**
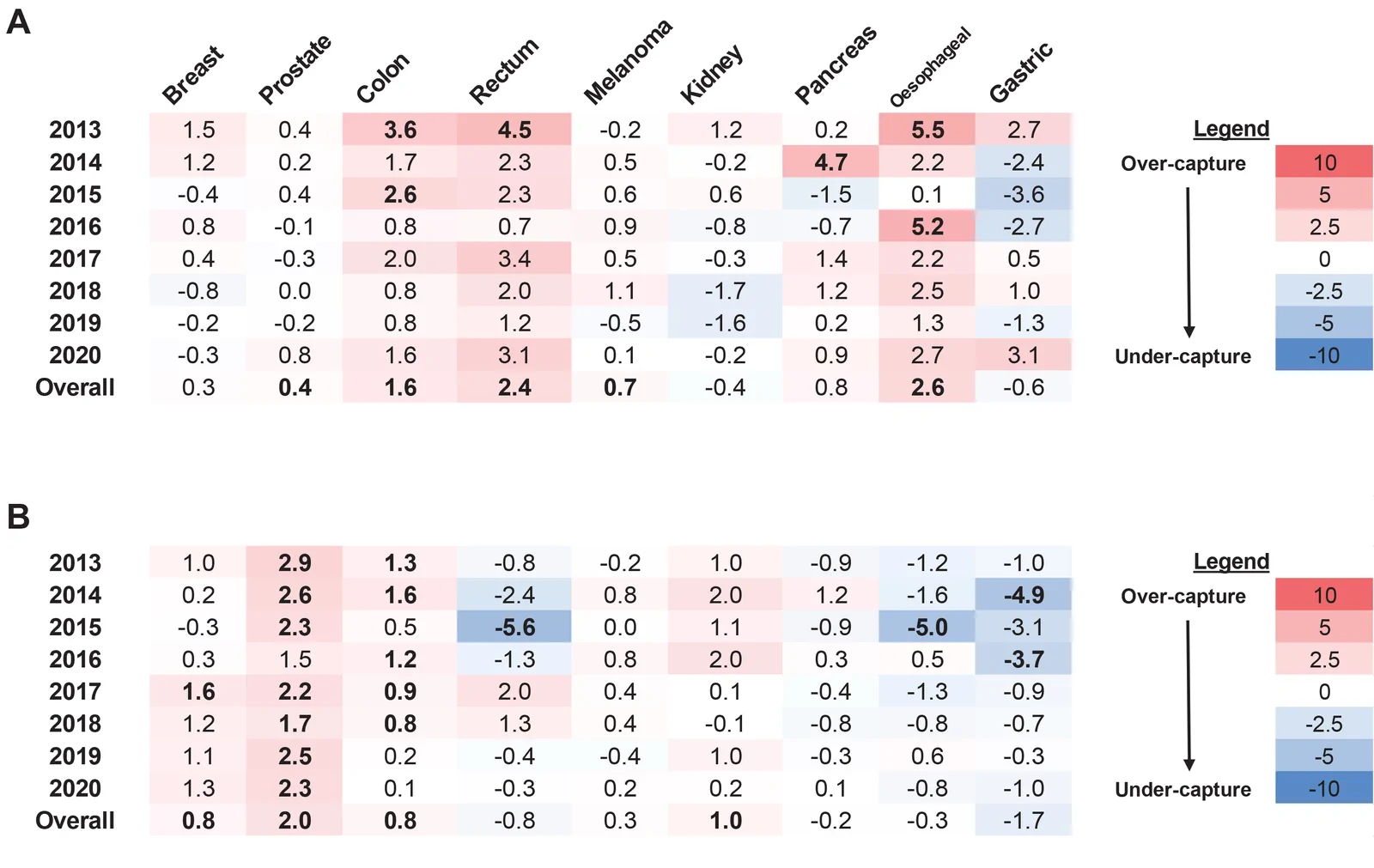
**Absolute percentage difference in treated proportions between the QResearch-linked dataset and NDRS national statistics, calendar years 2013-2020.** (A) SACT and (B) RT. Colours represent percentage-point difference in treated proportions (QResearch minus NDRS). Positive values indicate higher rates in QResearch; negative values indicate lower rates relative to NDRS. Bold values denote a significant difference, according to overlapping of confidence intervals. NDRS, National Disease Registration Service; QResearch, QResearch primary care database; RT, radiotherapy; SACT, systemic anticancer therapy.

Figure 3 shows the proportions of tumours treated with SACT and RT compared with NDRS national statistics. For SACT, proportions in the QResearch-linked dataset closely matched national statistics across sites. Across years, absolute differences were generally within ±3 percentage points, with the closest alignment observed in breast, prostate and kidney cancers and largest deviations seen in oesophageal and rectal cancers (Figure 4A).

Temporal patterns reflected increasing completeness of the SACT dataset. Before 2017, HES and NCRD data contributed a greater proportion of SACT ascertainment, compensating for incomplete SACT dataset submissions as residual differences versus NDRS remained small (Supplementary Figure S1 and Table S3, https://doi.org/10.1016/j.esmorw.2026.100763). Across most cancer types, the proportion of treatments identified through SACT dataset records increased over time, with stepwise improvements from 2014 onwards, consistent with the introduction of nationally mandated reporting.^8^

#### RT

RT-treated proportions in the linked dataset showed close agreement with NDRS national statistics for all cancer types (Figure 3B). Across the study period, overall differences were typically ≤2 percentage points (Figure 4B). The largest absolute difference was observed in prostate cancer, in which the QResearch-linked cohort showed a modestly higher proportion of RT-treated tumours (∼+2 percentage points) compared with national statistics. Source contribution analyses (Supplementary Figure S2 and Table S4, available at https://doi.org/10.1016/j.esmorw.2026.100763) highlighted that HES OPCS codes alone substantially undercaptured RT.

### Ascertainment of cancer cases without the NCRD

Among the 207 351 cancer records diagnosed between 2013 and 2020 across all sources, 194 442 (93.8%) were ascertained through QResearch, hospital or death registration data without reliance on the NCRD (Supplementary Figure S3A, available at https://doi.org/10.1016/j.esmorw.2026.100763). The remaining 12 909 tumours (6.2%) were identified only in the NCRD. When limiting analyses to first cancers only (*n* = 187 292), 96.0% of cases remained identifiable without registry linkage, with NCRD-only cases accounting for 4.0% (Supplementary Figure S3B, available at https://doi.org/10.1016/j.esmorw.2026.100763).

NCRD-only tumours were more frequently diagnosed in older adults and were earlier-stage disease (Table 2). They also included a higher proportion of melanoma and kidney cancers and a lower proportion of prostate cancers. They were substantially less likely to have recorded SACT or RT treatment, consistent with their predominance of earlier-stage disease. Patients with later stage disease are more likely to receive SACT or RT and hence more likely to be captured in hospital-based data sources. Sex, ethnicity and deprivation distributions were broadly similar between NCRD-only and other cases.

**Table 2:** Demographic and clinical characteristics of NCRD-only identified cancers compared with all other linked cases, England, calendar years 2013-2020

Demographics	NCRD-only patients, *n* (%)	Non-NCRD-only patients, *n* (%)	All eligible patients, *n* (%)
Total cancers (row %)	12 909 (6.2)	194 442 (93.8)	207 351
Cancer type			
Breast	3420 (26.5)	50 534 (26.0)	53 954 (26.0)
Prostate	1756 (13.6)	49 275 (25.3)	51 031 (24.6)
Colorectal	2913 (22.6)	38 588 (19.9)	41 501 (20.0)
Melanoma	1846 (14.3)	17 512 (9.0)	19 358 (9.3)
Kidney	1925 (14.9)	12 310 (6.3)	14 235 (6.9)
Pancreatic	460 (3.6)	11 035 (5.7)	11 495 (5.5)
Gastro-oesophageal	589 (4.6)	15 188 (7.8)	15 777 (7.6)
Sex			
Male	6070 (53.0)	103 710 (53.3)	109 780 (52.9)
Female	6839 (47.0)	90 732 (46.7)	97 571 (47.1)
Age at diagnosis (years)			
<50	1110 (8.6)	17 602 (9.0)	18 712 (9.0)
50-59	1617 (12.5)	29 285 (15.1)	30 902 (14.9)
60-69	2706 (21.0)	49 261 (25.3)	51 967 (25.1)
70-79	3897 (30.2)	56 582 (29.1)	60 479 (29.2)
≥80	3579 (27.7)	41 712 (21.5)	45 291 (21.8)
Year of diagnosis			
2013	1173 (9.1)	19 014 (9.8)	20 187 (9.7)
2014	1280 (9.9)	20 237 (10.4)	21 517 (10.4)
2015	1290 (10.0)	22 507 (11.6)	23 797 (11.5)
2016	1557 (12.1)	23 521 (12.1)	25 078 (12.1)
2017	1626 (12.6)	25 521 (13.1)	27 147 (13.1)
2018	1739 (13.5)	28 035 (14.4)	29 774 (14.4)
2019	2125 (16.5)	29 271 (15.1)	31 396 (15.1)
2020	2119 (16.4)	26 336 (13.5)	28 455 (13.7)
Region			
East Midlands	217 (1.7)	3746 (1.9)	3963 (1.9)
East of England	537 (4.2)	8785 (4.5)	9322 (4.5)
London	2264 (17.5)	32 785 (16.9)	35 049 (16.9)
North East	323 (2.5)	5451 (2.8)	5774 (2.8)
North West	2683 (20.8)	42 488 (21.9)	45 171 (21.8)
South East	3565 (27.6)	52 487 (27.0)	56 052 (27.0)
South West	1492 (11.6)	20 358 (10.5)	21 850 (10.5)
West Midlands	1463 (11.3)	22 514 (11.6)	23 977 (11.6)
Yorkshire and Humber	365 (2.8)	5828 (3.0)	6193 (3.0)
Ethnicity			
White	11 336 (92.5)	167 159 (92.2)	178 495 (92.2)
Asian	392 (3.2)	5414 (3.0)	5806 (3.0)
Black	268 (2.2)	4468 (2.5)	4736 (2.5)
Other	260 (2.1)	4267 (2.4)	4527 (2.3)
Unknown	653	13 134	13 787
Stage			
Unstageable	44 (0.5)	159 (0.1)	203 (0.1)
Stages 1 and 2	7165 (76.3)	87 426 (58.8)	94 591 (59.8)
Stages 3 and 4	2188 (23.3)	61 118 (41.1)	63 606 (40.0)
Unknown	3512	45 739	49 251
IMD quintile			
1 (most deprived)	1934 (15.0)	26 443 (15.4)	28 367 (15.3)
2	2354 (18.2)	31 711 (18.4)	34 065 (18.4)
3	2552 (19.8)	34 602 (20.1)	37 154 (20.1)
4	2826 (21.9)	37 622 (21.8)	40 448 (21.8)
5 (least deprived)	3243 (25.1)	41 886 (24.3)	45 129 (24.4)
Unknown	0 (0.0)	22 188	22 188
Treated proportions			
SACT-treated proportion (95% CI)	13.0 (12.4-13.7)	27.5 (27.3-27.7)	26.7 (26.5-26.9)
RT-treated proportion (95% CI)	14.4 (13.7-15.1)	31.7 (31.5-31.9)	30.8 (30.6-31.0)

Data represent records present in the NCRD only (‘registry-only’) versus those also identified in QResearch, HES or death records. Percentages calculated within known categories to allow for comparison. Values represent counts (%) unless otherwise indicated. Column percentages reported unless otherwise specified.

CI, confidence interval; HES, hospital episode statistics; IMD, index of multiple deprivation; NCRD, National Cancer Registration Dataset; QResearch, QResearch primary care database; RT, radiotherapy; SACT, systemic anticancer therapy.

Excluding NCRD-only cases, used here to approximate analyses conducted in which NCRD data are unavailable, had negligible impact on the overall demographic structure, cancer site and stage distributions, or treatment proportions. Results were similar in the first-cancer sensitivity analysis (Supplementary Table S5, available at https://doi.org/10.1016/j.esmorw.2026.100763).

## Discussion

### Summary of principal findings

In this population-based linked EHR data validation study, the QResearch-linked cancer dataset was highly representative of national cancer registrations and treatment data for seven major cancer sites. The cohort captured ∼15% of all incident cancers in England for these sites, with increasing coverage over time and inclusion of all English regions. Age, sex, stage, ethnicity and deprivation distributions closely aligned with national statistics, supporting the linked dataset’s generalisability.

Proportions of patients receiving SACT and RT closely matched national statistics over 2013-2020, with differences typically within a few percentage points across sites. Multisource treatment capture was important for maintaining concordance, particularly in the early study years.

Most cases (93.8% overall and 96.0% of first cancers) were ascertained without reliance on the cancer registry. NCRD-only cases comprised a small minority and were older, had earlier-stage disease, and were less likely to receive SACT or RT. Excluding these records, as a proxy for analyses conducted without the NCRD, had minimal impact on cohort composition and treatment distributions. Taken together, these findings support the validity of the QResearch-linked dataset for population oncology research.

### Interpretation of findings

Our findings support the accuracy of treatment indicators derived from linked EHR sources. The modest SACT overcapture in oesophageal and rectal cancers reflects the challenges of identifying complex multimodality regimens within routine data.^32^^,^^33^

For RT, reliance on hospital procedure coding alone substantially underestimated RT receipt compared with NDRS data, reflecting the outpatient and day-case delivery of many RT courses, which are known to be less complete than inpatient episodes.^26^^,^^34^ Our findings reinforce the importance of multisource treatment data for accurate RT capture using feeds such as the RTDS^10^ or the NCRD^6^ as utilised in this study.

Early SACT dataset submissions were incomplete, as noted in prior studies.^8^^,^^12^^,^^13^ Because estimation of survival following systemic therapy depends on accurate treatment start dates, and registry treatment indicators are undated, HES data provide important additional information for identifying treatment initiation in the earlier study years. Similarly, some oral anticancer therapies, particularly endocrine therapies prescribed in primary care, are not reliably captured within the SACT dataset.^35^ Primary care records often capture these treatments, representing an additional strength of the QResearch-linked resource.^35^

### Integration with previous literature

Our findings for analyses of NCRD and SACT are consistent with their published profiles, which emphasise near population coverage and evolving completeness.^6^^,^^8^^,^^12^ Previous UK and international linkage studies have largely explored case ascertainment and concordance of diagnoses across datasets, generally demonstrating high concordance between primary care and registry sources, with positive predictive values exceeding 90% for common cancers.^36^, ^37^, ^38^ However, completeness of primary care recording varies by site, with 9%-32% of registry-confirmed cases absent from primary care records, dependent on cancer type.^39^, ^40^, ^41^ Discrepancies have primarily been confined to diagnosis dates and site-specific coding variation.^12^^,^^39^^,^^41^^,^^42^ Our finding that 93.8% of cases were identifiable without the NCRD is broadly consistent with these estimates. However, the representativeness of a fully integrated primary care-hospital-registry-treatment linked dataset has been less well characterised, particularly with respect to demographic and treatment characteristics at a national level. Our validation approach demonstrates that such a dataset can reproduce national statistics with high fidelity when aligned to registry methodologies.

### Strengths and limitations

This study leverages one of the largest national primary care-hospital-registry linkages for oncology in England, capturing nearly 15% of incident cancers across seven major sites. Alignment of case definitions and treatment windows with national methodology allowed direct comparison with NDRS statistics, whereas source contribution and NCRD-only case analyses assessed robustness.

However, several limitations should be acknowledged. Analyses were restricted to seven common cancer types with matched ICD-10 definitions to the NDRS and may not generalise to rarer tumours. National comparator data for ethnicity, stage and deprivation were available from 2019 to 2020 rapid registration outputs with lower completeness.^30^

Outside the cancer registry, most data sources are patient-level rather than tumour-level, necessitating exclusion of patients with multiple cancers within 18 months. Coding quality and completeness in HES vary across trusts, although this did not materially affect treatment capture here.

Although excluding NCRD-only tumours had minimal impact on cohort representativeness, analyses conducted without NCRD data would have substantially reduced tumour-level information. For patients not receiving SACT, nonregistry sources generally provide cancer site and evidence of malignancy, but not detailed tumour characteristics such as stage or grade. Stage is recorded in the SACT dataset for a subset of SACT-treated patients but reflects stage at treatment initiation rather than diagnosis and has recognised data quality limitations.^8^ Analyses without NCRD data would also lack registry-derived treatment flags. Accordingly, although the present study supports the representativeness of the linked dataset, it does not constitute a validation of individual tumour-level variables across data sources.

Finally, we were unable to validate surgical resection proportions because the required variables were unavailable within our approved project.

### Implications for research

This validation supports the use of a QResearch-linked cancer dataset for real-world oncology research in England. Rich primary care data combined with robust capture of SACT and RT permits analyses of treatment selection, adherence, toxicity and outcomes, including the role of multimorbidity, polypharmacy and other lifestyle factors. The resource is therefore especially well-suited to pharmaco-epidemiological studies, analyses of time-varying exposures, and the development and external validation of risk prediction and prognostic models for cancer incidence and survival. This is consistent with international efforts to develop linked real-world oncology resources capable of evaluating treatment effectiveness, safety and outcomes at population scale.^43^, ^44^, ^45^

### QResearch data access and governance

The use of the QResearch-linked dataset is available for bona fide academic research in which there is a benefit to patients or the NHS.^46^ It is subject to governance, ethical and data access approvals, including application of NHS National Data Opt-Outs and access through secure research environments. Access is restricted to approved researchers working on approved projects under the oversight of the QResearch Scientific Committee and associated governance frameworks.^15^

### Conclusion

The QResearch-linked cancer dataset provides a robust, nationally representative platform for population oncology research in England. Demographic and treatment patterns align closely with national statistics and multisource linkage supports capture of SACT and RT. Finally, cancer cases identifiable without the NCRD remained highly representative, supporting the use of this dataset for high-quality real-world cancer research.

## References

[1] Hippisley-Cox J., Coupland C. (2017). Development and validation of risk prediction equations to estimate survival in patients with colorectal cancer: cohort study. BMJ.

[2] Hippisley-Cox J., Coupland C. (2015). Development and validation of risk prediction algorithms to estimate future risk of common cancers in men and women: prospective cohort study. BMJ Open.

[3] Bradley S.H., Lawrence N.R., Carder P. (2018). Using primary care data for health research in England—an overview. Future Healthc J.

[4] Thompson C.A., Jin A., Luft H.S. (2020). Population-based registry linkages to improve validity of electronic health record-based cancer research. Cancer Epidemiol Biomarkers Prev.

[5] Kanas G., Morimoto L., Mowat F., O’Malley C., Fryzek J., Nordyke R. (2010). Use of electronic medical records in oncology outcomes research. Clinicoecon Outcomes Res.

[6] Henson K.E., Elliss-Brookes L., Coupland V.H. (2020). Data resource profile: National Cancer Registration Dataset in England. Int J Epidemiol.

[7] Herbert A., Wijlaars L., Zylbersztejn A., Cromwell D., Hardelid P. (2017). Data resource profile: hospital episode statistics admitted patient care (HES APC). Int J Epidemiol.

[8] Bright C.J., Lawton S., Benson S. (2020). Data resource profile: the systemic anti-cancer therapy (SACT) dataset. Int J Epidemiol.

[9] Hippisley-Cox J., Stables D., Pringle M. (2004). QRESEARCH: a new general practice database for research. Inform Prim Care.

[10] Sandhu S., Sharpe M., Findlay Ú. (2023). Cohort profile: radiotherapy dataset (RTDS) in England. BMJ Open.

[11] Allemani C., Matsuda T., Di Carlo V. (2018). Global surveillance of trends in cancer survival 2000-14 (CONCORD-3): analysis of individual records for 37 513 025 patients diagnosed with one of 18 cancers from 322 population-based registries in 71 countries. Lancet.

[12] Gannon M.R., Park M.H., Miller K. (2023). Concordance of cancer drug therapy information derived from routinely collected hospital admissions data and the systemic anti-cancer therapy (SACT) dataset, for older women diagnosed with early invasive breast cancer in England. Cancer Epidemiol.

[13] McDonald L., Sammon C., Carroll R. (2020). Consistency of recording of chemotherapy cycles in the National Cancer Registration and Analysis Service Systemic Anti-Cancer Therapy database and the hospital episode statistics admitted patient care database. Future Oncol.

[14] NHS England (2025). Cancer Registrations Statistics, England. http://digital.nhs.uk/data-and-information/publications/statistical/cancer-registration-statistics.

[15] QResearch (2025). QResearch: Generating New Knowledge to Improve Patient Care. http://www.qresearch.org/.

[16] Hippisley-Cox J. (2013). https://www.qresearch.org/media/1055/completeness-and-validity-of-the-pseudonymised-nhs-number-in-qresearch-and-utility-for-data-linkage.pdf.

[17] Price G., Peek N., Eleftheriou I. (2025). An overview of real-world data infrastructure for cancer research. Clin Oncol.

[18] Hippisley-Cox J., Coupland C.A. (2025). Development and external validation of prediction algorithms to improve early diagnosis of cancer. Nat Commun.

[19] Clift A.K., Dodwell D., Lord S. (2023). Development and internal-external validation of statistical and machine learning models for breast cancer prognostication: cohort study. BMJ.

[20] Clift A.K., Tan P.S., Patone M. (2024). Predicting the risk of pancreatic cancer in adults with new-onset diabetes: development and internal-external validation of a clinical risk prediction model. Br J Cancer.

[21] Hippisley-Cox J., Mei W., Fitzgerald R., Coupland C. (2023). Development and validation of a novel risk prediction algorithm to estimate 10-year risk of oesophageal cancer in primary care: prospective cohort study and evaluation of performance against two other risk prediction models. Lancet Reg Health Eur.

[22] Hippisley-Cox J., Coupland C. (2013). Symptoms and risk factors to identify men with suspected cancer in primary care: derivation and validation of an algorithm. Br J Gen Pract.

[23] Hippisley-Cox J., Coupland C. (2013). Symptoms and risk factors to identify women with suspected cancer in primary care: derivation and validation of an algorithm. Br J Gen Pract.

[24] Hippisley-Cox J., Coupland C. (2011). Identifying women with suspected ovarian cancer in primary care: derivation and validation of algorithm. BMJ.

[25] Weiskopf N.G., Bakken S., Hripcsak G., Weng C. (2017). A data quality assessment guideline for electronic health record data reuse. eGEMs (Wash DC).

[26] Thorn J.C., Turner E.L., Hounsome L. (2016). Validation of the hospital episode statistics outpatient dataset in England. Pharmacoeconomics.

[27] NHS England (2025). https://digital.nhs.uk/services/data-access-request-service-dars/dars-products-and-services/data-set-catalogue/civil-registrations-of-death.

[28] National Disease Registration Service (2025). CAS-SOP 4v10 Linking Treatment Tables: Radiotherapy, SACT, and Tumour Resections. https://digital.nhs.uk/ndrs/data/data-outputs/cancer-data-hub/cancer-treatments.

[29] Møller H., Richards S., Hanchett N. (2011). Completeness of case ascertainment and survival time error in English cancer registries: impact on 1-year survival estimates. Br J Cancer.

[30] National Disease Registration Service (2025). Rapid Cancer Registration Data Set (RCRD). https://digital.nhs.uk/ndrs/data/data-sets/rcrd.

[31] NHS England (2025). NDRS Systemic Anti-Cancer Therapy Data Set (SACT). https://digital.nhs.uk/services/data-access-request-service-dars/dars-products-and-services/data-set-catalogue/ndrs-systemic-anti-cancer-therapy-data-set-sact#clinical-coding-systems.

[32] van Hagen P., Hulshof M.C.C.M., van Lanschot J.J.B. (2012). Preoperative chemoradiotherapy for esophageal or junctional cancer. N Engl J Med.

[33] Sebag-Montefiore D., Stephens R.J., Steele R. (2009). Preoperative radiotherapy versus selective postoperative chemoradiotherapy in patients with rectal cancer (MRC CR07 and NCIC-CTG C016): a multicentre, randomised trial. Lancet.

[34] NHS England (2013). NHS Hospital Data and Datasets: A Consultation. https://www.england.nhs.uk/wp-content/uploads/2013/07/hosp-data-consult.pdf.

[35] Gannon M.R., Dodwell D., Miller K. (2023). Completeness of endocrine therapy information in the primary care prescription database (PCPD) and secondary care treatment datasets: a national population-based cohort study using routine healthcare data. Cancer Epidemiol.

[36] Strongman H., Williams R., Bhaskaran K. (2020). What are the implications of using individual and combined sources of routinely collected data to identify and characterise incident site-specific cancers? A concordance and validation study using linked English electronic health records data. BMJ Open.

[37] Dregan A., Møller H., Murray-Thomas T., Gulliford M.C. (2012). Validity of cancer diagnosis in a primary care database compared with linked cancer registrations in England. Population-based cohort study. Cancer Epidemiol.

[38] Sollie A., Roskam J., Sijmons R.H., Numans M.E., Helsper C.W. (2016). Do GPs know their patients with cancer? Assessing the quality of cancer registration in Dutch primary care: a cross-sectional validation study. BMJ Open.

[39] Arhi C.S., Bottle A., Burns E.M. (2018). Comparison of cancer diagnosis recording between the Clinical Practice Research Datalink, Cancer Registry and Hospital Episodes Statistics. Cancer Epidemiol.

[40] Chaplin A.B., Archangelidi O., Hagberg K.W., Neasham D., Kafatos G. (2025). Comparison of epidemiological estimates for 19 cancer types using electronic health record databases in England: an analysis of CPRD aurum and CPRD GOLD databases with linked hospital episode statistics and cancer registry data. Clin Epidemiol.

[41] Whitfield E., White B., Barclay M.E. (2025). Differences in recording of cancer diagnosis between datasets in England: a population-based study of linked cancer registration, hospital, and primary care data. Cancer Epidemiol.

[42] Larsen I.K., Småstuen M., Johannesen T.B. (2009). Data quality at the Cancer Registry of Norway: an overview of comparability, completeness, validity and timeliness. Eur J Cancer.

[43] Penberthy L.T., Rivera D.R., Lund J.L., Bruno M.A., Meyer A.-M. (2022). An overview of real-world data sources for oncology and considerations for research. CA Cancer J Clin.

[44] Kuiper J.G., Herk-Sukel M.P.P., Lemmens V.E.P.P., Kuipers E.J., Herings R.M.C. (2022). A population-based linked cohort of cancer and primary care data: a new source to study the management of cancer in primary care. Eur J Cancer Care.

[45] Saesen R., Van Hemelrijck M., Bogaerts J. (2023). Defining the role of real-world data in cancer clinical research: the position of the European Organisation for Research and Treatment of Cancer. Eur J Cancer.

[46] QResearch (2026). Information for Researchers. https://www.qresearch.org/information/information-for-researchers/.

