# Supplementary material for: QResearch primary care records linked to national hospital and cancer registry data: a validation study
Source: ESMO Real World Data Digit Oncol. 2026 Aug 24;13:100763. doi: 10.1016/j.esmorw.2026.100763 (PMC13542614; doi:10.1016/j.esmorw.2026.100763)
Supplement: Supplementary file 1 [file mmc1.docx]

**SUPPLEMENTARY MATERIALS**

**Supplementary Table S1.** NDRS treatment attribution windows used to identify receipt of systemic anti-cancer therapy (SACT) and radiotherapy (RT) by cancer site.

**Supplementary Table S2 (A-G)**. Demographic characteristics by cancer type in the QResearch-linked dataset compared with NDRS national cancer registration statistics in England, calendar years 2013-2020.

**Supplementary Figure 1**. Incremental contribution of data sources to systemic anti-cancer therapy (SACT) ascertainment by cancer type and year (2013-2020).

**Supplementary Table S3 (A-I)**. Systemic anti-cancer therapy (SACT): QResearch-linked treated proportions and incremental source contributions by cancer type and year compared with NDRS data.

**Supplementary Figure 2**. Incremental contribution of data sources to radiotherapy (RT) ascertainment by cancer type and year (2013-2020).

**Supplementary Table S4 (A-I)**. Radiotherapy (RT): QResearch-linked treated proportion and incremental source contributions by cancer type and year compared with NDRS data.

**Supplementary Figure 3.** Distribution of cancer cases across linked data sources, England, 2013-2020. (**A**) Inclusive of all eligible cancer records diagnosed within study period, (**B**) sensitivity analysis limited to patients’ first cancer record only.

**Supplementary Table S5**. Demographic and clinical characteristics of registry-only identified first cancers compared with all other linked cases, England, calendar years 2013-2020.

**Supplementary Table S1.** NDRS treatment attribution windows used to identify receipt of systemic anti-cancer therapy (SACT) and radiotherapy (RT) by cancer site.

| **Cancer site and ICD-10 code** | **SACT attribution window (days from diagnosis)** | **RT attribution window (days from diagnosis)** |
| --- | --- | --- |
| **Breast (C50)** | 365 | 365 |
| **Prostate (C61)** | 365 | 365 |
| **Colorectal (C18-C20)** | 365 | 365 |
| **Melanoma (C43)** | 456 | 548 |
| **Kidney (C64-66, C68)** | 365 | 365 |
| **Pancreas (C25)** | 183 | 548 |
| **Gastro-oesophageal (C16-16)** | 183 | 274 |

*Footnote: Attribution windows are based on National Disease Registration Service (NDRS) methodology and define the period during which treatment is attributed to the incident cancer diagnosis. Windows extend from 31 days before diagnosis to the site- and modality-specific upper limit shown.*

*Abbreviations: NDRS - National Disease Registration Service; RT - radiotherapy; SACT - systemic anti-cancer therapy.*

**Supplementary Table S2 (A-G)**. Demographic characteristics by cancer type in the QResearch-linked dataset compared with NDRS national cancer registration statistics in England, calendar years 2013-2020.

*A – Breast cancer*

| **Demographics** | **QResearch**  **N (%)** | **NDRS**  **N (%)** |
| --- | --- | --- |
| **TOTAL CANCERS** | 50,832 | 366,009 |
| **SEX** |  |  |
| Male | 478 (0.9) | 2,560 (0.7) |
| Female | 50,354 (99.1) | 363,449 (99.3) |
| **AGE AT DIAGNOSIS (YEARS)** |  |  |
| <50 | 9,083 (17.9) | 67,783 (18.5) |
| 50-59 | 11,749 (23.1) | 80,846 (22.1) |
| 60-69 | 12,308 (24.2) | 89,043 (24.3) |
| 70-79 | 10,043 (19.8) | 69,927 (19.1) |
| 80+ | 7,649 (15.1) | 58,410 (16.0) |
| **YEAR OF DIAGNOSIS** |  |  |
| 2013 | 5,038 (11.2) | 44,831 (12.2) |
| 2014 | 5,449 (11.7) | 46,417 (12.7) |
| 2015 | 5,967 (12.9) | 46,083 (12.6) |
| 2016 | 6,211 (13.5) | 45,960 (12.6) |
| 2017 | 6,701 (14.5) | 46,109 (12.6) |
| 2018 | 7,303 (15.2) | 48,030 (13.1) |
| 2019 | 7,556 (15.6) | 48,387 (13.2) |
| 2020 | 6,607 (16.4) | 40,192 (11.0) |
| **REGION** |  |  |
| East Midlands | 950 (1.9) | 32,120 (8.8) |
| East of England | 2,258 (4.4) | 43,129 (11.8) |
| London | 9,015 (17.7) | 43,945 (12.0) |
| North East | 1,469 (2.9) | 18,077 (4.9) |
| North West | 11,040 (21.7) | 47,928 (13.1) |
| South East | 13,670 (26.9) | 65,628 (17.9) |
| South West | 5,174 (10.2) | 42,646 (11.7) |
| West Midlands | 5,751 (11.3) | 37,841 (10.3) |
| Yorkshire & Humber | 1,505 (3.0) | 34,695 (9.5) |
| **NB. BELOW DATA ONLY AVAILABLE FOR 2019-2020 (INCLUSIVE)** | 14,163 | 88,759 |
| **ETHNICITY** |  |  |
| White | 11,853 (89.1) | 73,956 (90.3) |
| Asian | 646 (4.9) | 3,534 (4.3) |
| Black | 323 (2.4) | 2,033 (2.5) |
| Other | 476 (3.6) | 2,295 (2.8) |
| Unknown | 865 | 2,715 |
| **STAGE** |  |  |
| Unstageable | 49 (0.4) | 230 (0.3) |
| Stage 1 & 2 | 9,600 (83.8) | 62,467 (84.0) |
| Stage 3 & 4 | 1,802 (15.7) | 11,666 (15.7) |
| Unknown | 2,712 | 14,216 |
| **IMD QUINTILE** |  |  |
| 1 (most deprived) | 2,040 (15.1) | 13,351 (15.1) |
| 2 | 2,588 (19.1) | 16,138 (18.2) |
| 3 | 2,715 (20.1) | 18,594 (21.0) |
| 4 | 2,893 (21.4) | 19,808 (22.4) |
| 5 (least deprived) | 3,304 (24.4) | 20,688 (23.4) |
| Unknown | 623 | 0 |

*B – Prostate cancer*

| **Demographics** | **QResearch**  **N (%)** | **NDRS**  **N (%)** |
| --- | --- | --- |
| **TOTAL CANCERS** | 47,790 | 335,439 |
| **AGE AT DIAGNOSIS (YEARS)** |  |  |
| <50 | 477 (1.0) | 3,748 (1.1) |
| 50-59 | 4,969 (10.4) | 37,122 (11.1) |
| 60-69 | 14,839 (31.1) | 109,125 (32.5) |
| 70-79 | 18,427 (38.6) | 126,362 (37.7) |
| 80+ | 9,078 (19.0) | 59,082 (17.6) |
| **YEAR OF DIAGNOSIS** |  |  |
| 2013 | 4,671 (11.6) | 40,372 (12.0) |
| 2014 | 4,727 (11.9) | 39,741 (11.8) |
| 2015 | 5,495 (13.6) | 40,331 (12.0) |
| 2016 | 5,680 (14.0) | 40,489 (12.1) |
| 2017 | 6,150 (14.9) | 41,201 (12.3) |
| 2018 | 7,364 (14.8) | 49,810 (14.8) |
| 2019 | 7,454 (15.7) | 47,479 (14.2) |
| 2020 | 6,249 (17.4) | 36,016 (10.7) |
| **REGION** |  |  |
| East Midlands | 982 (2.1) | 29,371 (8.8) |
| East of England | 2,376 (5.0) | 42,249 (12.6) |
| London | 8,566 (17.9) | 38,888 (11.6) |
| North East | 1,135 (2.4) | 14,729 (4.4) |
| North West | 9,332 (19.5) | 40,162 (12.0) |
| South East | 13,419 (28.1) | 62,645 (18.7) |
| South West | 4,954 (10.4) | 40,113 (12.0) |
| West Midlands | 5,630 (11.8) | 35,331 (10.5) |
| Yorkshire & Humber | 1,396 (2.9) | 31,951 (9.5) |
| **NB. BELOW DATA ONLY AVAILABLE FOR 2019-2020 (INCLUSIVE)** | 13,703 | 83,495 |
| **ETHNICITY** |  |  |
| White | 11,470 (90.2) | 68,014 (91.9) |
| Asian | 323 (2.5) | 1,689 (2.3) |
| Black | 524 (4.1) | 2,844 (3.8) |
| Other | 398 (3.1) | 1,428 (1.9) |
| Unknown | 988 | 2,865 |
| **STAGE** |  |  |
| Unstageable | 0 (0.0) | 102 (0.2) |
| Stage 1 & 2 | 5,711 (53.1) | 35,436 (52.2) |
| Stage 3 & 4 | 5,054 (46.9) | 32,348 (47.7) |
| Unknown | 2,938 | 15,609 |
| **IMD QUINTILE** |  |  |
| 1 (most deprived) | 1,699 (12.7) | 11,008 (13.2) |
| 2 | 2,346 (17.6) | 14,271 (17.1) |
| 3 | 2,834 (21.2) | 17,806 (21.3) |
| 4 | 2,983 (22.4) | 19,654 (23.5) |
| 5 (least deprived) | 3,476 (26.1) | 20,756 (24.9) |
| Unknown | 365 | 0 |

*C – Colorectal cancer*

| **Demographics** | **QResearch**  **N (%)** | **NDRS**  **N (%)** |
| --- | --- | --- |
| **TOTAL CANCERS** | 40,818 | 280,321 |
| **SEX** |  |  |
| Male | 22,615 (55.4) | 155,785 (55.6) |
| Female | 18,203 (44.6) | 124,536 (44.4) |
| **AGE AT DIAGNOSIS (YEARS)** |  |  |
| <50 | 2,423 (5.9) | 17,239 (6.1) |
| 50-59 | 4,554 (11.2) | 32,045 (11.4) |
| 60-69 | 9,316 (22.8) | 64,463 (23.0) |
| 70-79 | 12,558 (30.8) | 85,879 (30.6) |
| 80+ | 11,967 (29.3) | 80,695 (28.8) |
| **YEAR OF DIAGNOSIS** |  |  |
| 2013 | 3,998 (11.8) | 33,765 (12.0) |
| 2014 | 4,245 (12.5) | 34,025 (12.1) |
| 2015 | 4,641 (13.4) | 34,729 (12.4) |
| 2016 | 5,018 (14.4) | 34,952 (12.5) |
| 2017 | 5,286 (15.2) | 34,825 (12.4) |
| 2018 | 5,545 (15.4) | 35,958 (12.8) |
| 2019 | 6,134 (16.3) | 37,662 (13.4) |
| 2020 | 5,951 (17.3) | 34,405 (12.3) |
| **REGION** |  |  |
| East Midlands | 836 (2.1) | 25,162 (9.0) |
| East of England | 1,874 (4.6) | 33,038 (11.8) |
| London | 6,388 (15.7) | 28,887 (10.3) |
| North East | 1,218 (3.0) | 15,050 (5.4) |
| North West | 9,375 (23.0) | 38,669 (13.8) |
| South East | 10,571 (25.9) | 48,129 (17.2) |
| South West | 4,274 (10.5) | 33,087 (11.8) |
| West Midlands | 5,031 (12.3) | 30,767 (11.0) |
| Yorkshire & Humber | 1,251 (3.1) | 27,532 (9.8) |
| **NB. BELOW DATA ONLY AVAILABLE FOR 2019-2020 (INCLUSIVE)** | 12,085 | 72,067 |
| **ETHNICITY** |  |  |
| White | 10,607 (92.8) | 63,415 (93.7) |
| Asian | 365 (3.2) | 1,768 (2.6) |
| Black | 213 (1.9) | 1,205 (1.8) |
| Other | 239 (2.1) | 1,299 (1.9) |
| Unknown | 661 | 2,347 |
| **STAGE** |  |  |
| Unstageable | 0 (0.0) | 28 (0.0) |
| Stage 1 & 2 | 4,116 (43.1) | 26,678 (44.1) |
| Stage 3 & 4 | 5,433 (56.9) | 33,749 (55.8) |
| Unknown | 2,536 | 11,612 |
| **IMD QUINTILE** |  |  |
| 1 (most deprived) | 1,783 (15.6) | 11,493 (15.9) |
| 2 | 2,192 (19.2) | 13,051 (18.1) |
| 3 | 2,272 (19.9) | 15,248 (21.2) |
| 4 | 2,423 (21.2) | 16,093 (22.3) |
| 5 (least deprived) | 2,755 (24.1) | 16,182 (22.5) |
| Unknown | 660 | 0 |

*D – Melanoma*

| **Demographics** | **QResearch**  **N (%)** | **NDRS**  **N (%)** |
| --- | --- | --- |
| **TOTAL CANCERS** | 16,831 | 108,646 |
| **SEX** |  |  |
| Male | 8,547 (50.8) | 54,655 (50.3) |
| Female | 8,284 (49.2) | 53,991 (49.7) |
| **AGE AT DIAGNOSIS (YEARS)** |  |  |
| <50 | 3,313 (19.7) | 22,968 (21.1) |
| 50-59 | 2,763 (16.4) | 18,163 (16.7) |
| 60-69 | 3,496 (20.8) | 22,968 (21.1) |
| 70-79 | 4,099 (24.4) | 25,305 (23.3) |
| 80+ | 3,160 (18.8) | 19,242 (17.7) |
| **YEAR OF DIAGNOSIS** |  |  |
| 2013 | 1,536 (12.5) | 12,246 (11.3) |
| 2014 | 1,784 (13.7) | 12,993 (12.0) |
| 2015 | 1,893 (14.2) | 13,357 (12.3) |
| 2016 | 2,086 (15.2) | 13,748 (12.7) |
| 2017 | 2,286 (16.6) | 13,740 (12.6) |
| 2018 | 2,421 (16.3) | 14,824 (13.6) |
| 2019 | 2,597 (17.0) | 15,261 (14.0) |
| 2020 | 2,228 (17.9) | 12,477 (11.5) |
| **REGION** |  |  |
| East Midlands | 327 (1.9) | 8,803 (8.1) |
| East of England | 701 (4.2) | 12,393 (11.4) |
| London | 2,157 (12.8) | 9,208 (8.5) |
| North East | 527 (3.1) | 6,546 (6.0) |
| North West | 3,799 (22.6) | 15,137 (13.9) |
| South East | 5,076 (30.2) | 21,908 (20.2) |
| South West | 2,045 (12.2) | 15,423 (14.2) |
| West Midlands | 1,715 (10.2) | 9,697 (8.9) |
| Yorkshire & Humber | 484 (2.9) | 9,531 (8.8) |
| **NB. BELOW DATA ONLY AVAILABLE FOR 2019-2020 (INCLUSIVE)** | 4,825 | 27,738 |
| **ETHNICITY** |  |  |
| White | 4,349 (98.4) | 23,992 (98.4) |
| Asian | < 10 (< 0.3) | 64 (0.3) |
| Black | < 20 (< 0.5) | 64 (0.3) |
| Other | 46 (1.0) | 250 (1.0) |
| Unknown | 405 | 928 |
| **STAGE** |  |  |
| Unstageable | 0 (0.0) | 0 (0.0) |
| Stage 1 & 2 | 3,247 (87.9) | 21,135 (88.1) |
| Stage 3 & 4 | 446 (12.1) | 2,848 (11.9) |
| Unknown | 1,132 | 3,755 |
| **IMD QUINTILE** |  |  |
| 1 (most deprived) | 448 (10.5) | 2,962 (10.7) |
| 2 | 632 (14.9) | 4,237 (15.3) |
| 3 | 845 (19.9) | 5,796 (20.9) |
| 4 | 1,056 (24.9) | 6,866 (24.8) |
| 5 (least deprived) | 1,267 (29.8) | 7,877 (28.4) |
| Unknown | 577 | 0 |

*Footnote. Ethnicity values suppressed as counts <10 for some subgroups.*

*E – Kidney cancer*

| **Demographics** | **QResearch**  **N (%)** | **NDRS**  **N (%)** |
| --- | --- | --- |
| **TOTAL CANCERS** | 13,793 | 85,884 |
| **SEX** |  |  |
| Male | 8,830 (64.0) | 54,367 (63.3) |
| Female | 4,963 (36.0) | 31,517 (36.7) |
| **AGE AT DIAGNOSIS (YEARS)** |  |  |
| <50 | 1,003 (7.3) | 8,160 (9.5) |
| 50-59 | 1,961 (14.2) | 13,169 (15.3) |
| 60-69 | 3,385 (24.5) | 21,376 (24.9) |
| 70-79 | 4,312 (31.3) | 24,906 (29.0) |
| 80+ | 3,132 (22.7) | 18,273 (21.3) |
| **YEAR OF DIAGNOSIS** |  |  |
| 2013 | 1,345 (13.6) | 9,900 (11.5) |
| 2014 | 1,484 (14.1) | 10,517 (12.2) |
| 2015 | 1,560 (14.8) | 10,507 (12.2) |
| 2016 | 1,642 (15.4) | 10,690 (12.4) |
| 2017 | 1,760 (16.4) | 10,759 (12.5) |
| 2018 | 1,897 (17.2) | 11,031 (12.8) |
| 2019 | 2,052 (17.4) | 11,827 (13.8) |
| 2020 | 2,053 (19.3) | 10,653 (12.4) |
| **REGION** |  |  |
| East Midlands | 215 (1.6) | 6,936 (8.1) |
| East of England | 590 (4.3) | 9,556 (11.1) |
| London | 2,078 (15.1) | 8,983 (10.5) |
| North East | 451 (3.3) | 5,024 (5.8) |
| North West | 3,251 (23.6) | 12,255 (14.3) |
| South East | 3,657 (26.5) | 14,419 (16.8) |
| South West | 1,584 (11.5) | 10,267 (12.0) |
| West Midlands | 1,560 (11.3) | 8,790 (10.2) |
| Yorkshire & Humber | 407 (3.0) | 9,654 (11.2) |
| **NB. BELOW DATA ONLY AVAILABLE FOR 2019-2020 (INCLUSIVE)** | 4,105 | 22,480  (Stage 19,229) |
| **ETHNICITY** |  |  |
| White | 3,614 (92.8) | 15,523 (92.3) |
| Asian | 137 (3.5) | 587 (3.5) |
| Black | 71 (1.8) | 341 (2.0) |
| Other | 74 (1.9) | 375 (2.2) |
| Unknown | 209 | 526 |
| **STAGE** |  |  |
| Unstageable | 0 (0.0) | 27 (0.2) |
| Stage 1 & 2 | 1,367 (53.5) | 7,976 (56.2) |
| Stage 3 & 4 | 1,190 (46.5) | 6,186 (43.6) |
| Unknown | 1,548 | 5,040 |
| **IMD QUINTILE** |  |  |
| 1 (most deprived) | 622 (17.7) | 4,056 (18.0) |
| 2 | 729 (20.7) | 4,321 (19.2) |
| 3 | 698 (19.8) | 4,658 (20.7) |
| 4 | 694 (19.7) | 4,462 (19.8) |
| 5 (least deprived) | 775 (22.0) | 4,684 (20.8) |
| Unknown | 587 | 0 |

*Footnote. Stage data only available for ICD code C64 (not C65, C66 or C68).*

*F – Pancreatic cancer*

| **Demographics** | **QResearch**  **N (%)** | **NDRS**  **N (%)** |
| --- | --- | --- |
| **TOTAL CANCERS** | 11,077 | 69,086 |
| **SEX** |  |  |
| Male | 5,665 (51.1) | 35,332 (51.1) |
| Female | 5,412 (48.9) | 33,754 (48.9) |
| **AGE AT DIAGNOSIS (YEARS)** |  |  |
| <50 | 422 (3.8) | 2,666 (4.1) |
| 50-59 | 1,108 (10.0) | 7,047 (10.2) |
| 60-69 | 2,433 (22.0) | 15,462 (22.4) |
| 70-79 | 3,682 (33.2) | 22,598 (32.7) |
| 80+ | 3,432 (31.0) | 21,313 (30.8) |
| **YEAR OF DIAGNOSIS** |  |  |
| 2013 | 1,056 (13.4) | 7,887 (11.4) |
| 2014 | 1,117 (13.8) | 8,080 (11.7) |
| 2015 | 1,274 (15.3) | 8,319 (12.0) |
| 2016 | 1,287 (15.2) | 8,455 (12.2) |
| 2017 | 1,541 (17.5) | 8,829 (12.8) |
| 2018 | 1,481 (16.7) | 8,885 (12.9) |
| 2019 | 1,619 (17.5) | 9,263 (13.4) |
| 2020 | 1,702 (18.2) | 9,368 (13.6) |
| **REGION** |  |  |
| East Midlands | 193 (1.7) | 6,111 (8.8) |
| East of England | 489 (4.4) | 8,160 (11.8) |
| London | 1,944 (17.6) | 7,523 (10.9) |
| North East | 289 (2.6) | 3,574 (5.2) |
| North West | 2,367 (21.4) | 8,900 (12.9) |
| South East | 2,980 (26.9) | 12,311 (17.8) |
| South West | 1,196 (10.8) | 8,247 (11.9) |
| West Midlands | 1,282 (11.6) | 7,290 (10.6) |
| Yorkshire & Humber | 337 (3.0) | 6,970 (10.1) |
| **NB. BELOW DATA ONLY AVAILABLE FOR 2019-2020 (INCLUSIVE)** | 3,321 | 18,631 |
| **ETHNICITY** |  |  |
| White | 2,898 (92.1) | 15,442 (93.4) |
| Asian | 89 (2.8) | 440 (2.7) |
| Black | 78 (2.5) | 335 (2.0) |
| Other | 83 (2.6) | 323 (2.0) |
| Unknown | 173 | 689 |
| **STAGE** |  |  |
| Unstageable | 0 (0.0) | < 10 (< 0.1) |
| Stage 1 & 2 | 526 (24.2) | < 3,220 (< 23.9) |
| Stage 3 & 4 | 1,645 (75.8) | 10,281 (76.2) |
| Unknown | 1,150 | 5,134 |
| **IMD QUINTILE** |  |  |
| 1 (most deprived) | 509 (16.8) | 3,115 (16.7) |
| 2 | 585 (19.4) | 3,538 (19.0) |
| 3 | 620 (20.5) | 3,898 (20.9) |
| 4 | 631 (20.9) | 4,125 (22.1) |
| 5 (least deprived) | 677 (22.4) | 3,955 (21.2) |
| Unknown | 299 | 0 |

*G – Gastro-oesophageal cancer*

| **Demographics** | **QResearch**  **N (%)** | **NDRS**  **N (%)** |
| --- | --- | --- |
| **TOTAL CANCERS** | 15,424 | 102,858 |
| **SEX** |  |  |
| Male | 10,328 (67.0) | 69,309 (67.4) |
| Female | 5,096 (33.0) | 33,549 (32.6) |
| **AGE AT DIAGNOSIS (YEARS)** |  |  |
| <50 | 627 (4.1) | 4,414 (4.3) |
| 50-59 | 1,734 (11.2) | 11,563 (11.2) |
| 60-69 | 3,633 (23.6) | 24,005 (23.3) |
| 70-79 | 4,980 (32.3) | 33,006 (32.1) |
| 80+ | 4,450 (28.9) | 29,870 (29.0) |
| **YEAR OF DIAGNOSIS** |  |  |
| 2013 | 1,627 (12.6) | 12,890 (12.5) |
| 2014 | 1,693 (13.4) | 12,656 (12.3) |
| 2015 | 1,786 (13.6) | 13,110 (12.7) |
| 2016 | 1,896 (14.7) | 12,875 (12.5) |
| 2017 | 1,950 (15.3) | 12,712 (12.4) |
| 2018 | 2,091 (16.2) | 12,925 (12.6) |
| 2019 | 2,207 (16.4) | 13,419 (13.0) |
| 2020 | 2,174 (17.7) | 12,271 (11.9) |
| **REGION** |  |  |
| East Midlands | 289 (1.9) | 9,903 (9.6) |
| East of England | 594 (3.9) | 11,277 (11.0) |
| London | 2,492 (16.2) | 10,409 (10.1) |
| North East | 497 (3.2) | 5,969 (5.8) |
| North West | 3,904 (25.3) | 15,709 (15.3) |
| South East | 3,584 (23.2) | 15,381 (15.0) |
| South West | 1,484 (9.6) | 10,848 (10.5) |
| West Midlands | 2,062 (13.4) | 12,133 (11.8) |
| Yorkshire & Humber | 518 (3.4) | 11,229 (10.9) |
| **NB. BELOW DATA ONLY AVAILABLE FOR 2019-2020 (INCLUSIVE)** | 4,381 | 25,690  (Stage 18,255) |
| **ETHNICITY** |  |  |
| White | 3,835 (91.9) | 21,866 (93.5) |
| Asian | 158 (3.8) | 633 (2.7) |
| Black | 86 (2.1) | 473 (2.0) |
| Other | 96 (2.3) | 409 (1.7) |
| Unknown | 206 | 794 |
| **STAGE** |  |  |
| Unstageable | 0 (0.0) | 32 (0.2) |
| Stage 1 & 2 | 415 (18.4) | 2,599 (19.1) |
| Stage 3 & 4 | 1835 (81.6) | 10,954 (80.6) |
| Unknown | 851 | 4,670 |
| **IMD QUINTILE** |  |  |
| 1 (most deprived) | 866 (20.9) | 5,151 (20.1) |
| 2 | 806 (19.5) | 5,131 (20.0) |
| 3 | 837 (20.2) | 5,250 (20.4) |
| 4 | 820 (19.8) | 5,318 (20.7) |
| 5 (least deprived) | 805 (19.5) | 4,840 (18.8) |
| Unknown | 247 | 0 |

*Footnote. Stage only includes C15 and C16.0 (oesophageal cancer only).*

**Supplementary Figure 1**. Incremental contribution of data sources to systemic anti-cancer therapy (SACT) ascertainment by cancer type and year (2013-2020).

*Footnote: Bars show cumulative treatment ascertainment. Green represents cases identified in the SACT dataset; yellow represents additional cases identified through Hospital Episode Statistics (HES); and purple represents further cases identified through the cancer registry (NCRD). The red line indicates the corresponding NDRS treated proportion. Categories are mutually exclusive within each stack (i.e. additional contributions represent cases not previously identified in preceding sources).*

*Abbreviations: HES – Hospital Episode Statistics; NCRD – National Cancer Registration Dataset; NDRS – National Disease Registration Service; SACT – systemic anti-cancer therapy dataset.*

**Supplementary Table S3 (A-I)**. Systemic anti-cancer therapy (SACT): QResearch-linked treated proportions and incremental source contributions by cancer type and year compared with NDRS data.

*A – Breast cancer*

|  | **SACT database only SACT proportion, % (95% CI)** | **SACT database & HES SACT proportion, % (95% CI)** | **SACT database & HES & Cancer registry SACT proportion, % (95% CI)** | **NDRS SACT proportion, % (95% CI)** |
| --- | --- | --- | --- | --- |
| **2013** | 32.8 (31.5 - 34.2) | 36.1 (34.7 - 37.5) | 37.2 (35.8 - 38.6) | 35.7 (35.3 - 36.2) |
| **2014** | 32.6 (31.3 - 33.9) | 35.0 (33.7 - 36.3) | 36.1 (34.8 - 37.5) | 34.9 (34.5 - 35.3) |
| **2015** | 30.7 (29.5 - 32.0) | 33.0 (31.7 - 34.2) | 34.2 (33.0 - 35.5) | 34.6 (34.2 - 35.0) |
| **2016** | 31.9 (30.7 - 33.1) | 34.7 (33.5 - 35.9) | 36.5 (35.2 - 37.7) | 35.7 (35.2 - 36.1) |
| **2017** | 32.1 (30.9 - 33.2) | 34.0 (32.8 - 35.1) | 36.9 (35.7 - 38.1) | 36.4 (36.0 - 36.9) |
| **2018** | 30.8 (29.7 - 31.9) | 32.0 (30.9 - 33.1) | 35.1 (34.0 - 36.3) | 35.9 (35.5 - 36.3) |
| **2019** | 31.3 (30.2 - 32.4) | 32.8 (31.6 - 33.9) | 35.7 (34.5 - 36.8) | 35.8 (35.4 - 36.3) |
| **2020** | 32.9 (31.7 - 34.1) | 34.5 (33.3 - 35.7) | 37.0 (35.8 - 38.2) | 37.4 (36.9 - 37.8) |
| **Overall** | 31.8 (31.4 - 32.2) | 33.9 (33.4 - 34.3) | 36.1 (35.6 - 36.5) | 35.8 (35.6 - 35.9) |

*B – Prostate cancer*

|  | **SACT database only SACT proportion, % (95% CI)** | **SACT database & HES SACT proportion, % (95% CI)** | **SACT database & HES & Cancer registry SACT proportion, % (95% CI)** | **NDRS SACT proportion, % (95% CI)** |
| --- | --- | --- | --- | --- |
| **2013** | 1.9 (1.5 - 2.4) | 3.1 (2.5 - 3.6) | 4.5 (3.8 - 5.1) | 4.0 (3.9 - 4.2) |
| **2014** | 2.1 (1.7 - 2.5) | 3.1 (2.6 - 3.6) | 4.1 (3.5 - 4.7) | 3.9 (3.7 - 4.1) |
| **2015** | 4.7 (4.2 - 5.3) | 5.9 (5.3 - 6.6) | 6.8 (6.1 - 7.5) | 6.4 (6.2 - 6.6) |
| **2016** | 6.8 (6.1 - 7.5) | 7.8 (7.1 - 8.5) | 8.5 (7.8 - 9.3) | 8.6 (8.3 - 8.9) |
| **2017** | 7.7 (7.0 - 8.4) | 8.5 (7.8 - 9.2) | 9.1 (8.4 - 9.9) | 9.5 (9.2 - 9.8) |
| **2018** | 7.5 (6.9 - 8.2) | 8.5 (7.8 - 9.1) | 8.9 (8.2 - 9.6) | 8.9 (8.7 - 9.2) |
| **2019** | 7.3 (6.7 - 7.9) | 8.1 (7.5 - 8.8) | 8.5 (7.9 - 9.2) | 8.8 (8.5 - 9.0) |
| **2020** | 10.0 (9.2 - 10.8) | 11.1 (10.3 - 11.9) | 12.0 (11.2 - 12.8) | 11.2 (10.9 - 11.5) |
| **Overall** | 6.3 (6.1 - 6.6) | 7.3 (7.1 - 7.6) | 8.1 (7.8 - 8.3) | 7.7 (7.6 - 7.8) |

*C – Colon cancer*

|  | **SACT database only SACT proportion, % (95% CI)** | **SACT database & HES SACT proportion, % (95% CI)** | **SACT database & HES & Cancer registry SACT proportion, % (95% CI)** | **NDRS SACT proportion, % (95% CI)** |
| --- | --- | --- | --- | --- |
| **2013** | 28.5 (26.7 - 30.3) | 33.8 (31.9 - 35.6) | 35.2 (33.3 - 37.1) | 31.7 (31.1 - 32.2) |
| **2014** | 30.2 (28.4 - 31.9) | 32.7 (30.9 - 34.5) | 33.6 (31.8 - 35.4) | 31.9 (31.3 - 32.5) |
| **2015** | 30.2 (28.5 - 31.8) | 33.3 (31.6 - 35.0) | 34.6 (32.8 - 36.3) | 32.0 (31.4 - 32.5) |
| **2016** | 30.2 (28.6 - 31.8) | 31.5 (29.9 - 33.1) | 32.5 (30.9 - 34.2) | 31.8 (31.2 - 32.4) |
| **2017** | 31.5 (30.0 - 33.1) | 33.0 (31.4 - 34.6) | 34.2 (32.5 - 35.8) | 32.1 (31.6 - 32.7) |
| **2018** | 30.6 (29.1 - 32.1) | 31.7 (30.2 - 33.2) | 32.7 (31.1 - 34.2) | 31.9 (31.3 - 32.4) |
| **2019** | 30.1 (28.7 - 31.5) | 30.9 (29.5 - 32.4) | 31.8 (30.4 - 33.3) | 31.0 (30.5 - 31.6) |
| **2020** | 30.0 (28.5 - 31.4) | 31.0 (29.5 - 32.4) | 31.6 (30.1 - 33.0) | 29.9 (29.4 - 30.5) |
| **Overall** | 30.2 (29.6 - 30.8) | 32.1 (31.5 - 32.7) | 33.1 (32.5 - 33.7) | 31.5 (31.3 - 31.7) |

*D – Rectal cancer*

|  | **SACT database only SACT proportion, % (95% CI)** | **SACT database & HES SACT proportion, % (95% CI)** | **SACT database & HES & Cancer registry SACT proportion, % (95% CI)** | **NDRS SACT proportion, % (95% CI)** |
| --- | --- | --- | --- | --- |
| **2013** | 38.8 (35.8 - 41.8) | 44.2 (41.1 - 47.2) | 47.5 (44.4 - 50.6) | 43.0 (42.0 - 44.0) |
| **2014** | 39.7 (36.7 - 42.6) | 42.6 (39.6 - 45.6) | 45.7 (42.7 - 48.7) | 43.4 (42.4 - 44.4) |
| **2015** | 39.3 (36.4 - 42.2) | 42.7 (39.8 - 45.7) | 45.8 (42.9 - 48.8) | 43.5 (42.5 - 44.5) |
| **2016** | 40.4 (37.6 - 43.2) | 42.4 (39.6 - 45.2) | 44.3 (41.5 - 47.1) | 43.6 (42.6 - 44.6) |
| **2017** | 43.7 (41.0 - 46.5) | 45.6 (42.9 - 48.4) | 47.4 (44.7 - 50.2) | 44.1 (43.1 - 45.1) |
| **2018** | 44.2 (41.5 - 46.9) | 45.3 (42.6 - 48.0) | 46.4 (43.7 - 49.1) | 44.3 (43.4 - 45.3) |
| **2019** | 41.9 (39.4 - 44.5) | 43.1 (40.6 - 45.6) | 45.2 (42.6 - 47.7) | 43.9 (43.0 - 44.9) |
| **2020** | 42.5 (39.9 - 45.1) | 43.9 (41.2 - 46.5) | 45.5 (42.8 - 48.1) | 42.4 (41.4 - 43.4) |
| **Overall** | 41.5 (40.5 - 42.5) | 43.8 (42.8 - 44.7) | 45.9 (44.9 - 46.9) | 43.6 (43.2 - 43.9) |

*E – Melanoma*

|  | **SACT database only SACT proportion, % (95% CI)** | **SACT database & HES SACT proportion, % (95% CI)** | **SACT database & HES & Cancer registry SACT proportion, % (95% CI)** | **NDRS SACT proportion, % (95% CI)** |
| --- | --- | --- | --- | --- |
| **2013** | 2.7 (1.8 - 3.5) | 3.2 (2.3 - 4.1) | 3.4 (2.4 - 4.3) | 3.6 (3.3 - 4.0) |
| **2014** | 3.9 (3.0 - 4.9) | 4.2 (3.2 - 5.2) | 4.5 (3.5 - 5.5) | 4.0 (3.7 - 4.3) |
| **2015** | 4.7 (3.7 - 5.7) | 5.4 (4.4 - 6.5) | 5.6 (4.5 - 6.7) | 5.0 (4.7 - 5.4) |
| **2016** | 5.4 (4.4 - 6.4) | 6.2 (5.1 - 7.3) | 6.3 (5.2 - 7.4) | 5.4 (5.1 - 5.8) |
| **2017** | 5.8 (4.8 - 6.8) | 6.5 (5.4 - 7.6) | 6.5 (5.5 - 7.6) | 6.1 (5.7 - 6.5) |
| **2018** | 9.0 (7.8 - 10.2) | 9.8 (8.5 - 11.1) | 10.1 (8.9 - 11.4) | 9.0 (8.5 - 9.4) |
| **2019** | 10.2 (9.0 - 11.5) | 10.9 (9.6 - 12.2) | 10.9 (9.6 - 12.2) | 11.4 (10.9 - 12.0) |
| **2020** | 12.6 (11.1 - 14.0) | 13.3 (11.8 - 14.8) | 13.6 (12.1 - 15.1) | 13.5 (12.9 - 14.1) |
| **Overall** | 7.2 (6.8 - 7.6) | 7.8 (7.4 - 8.3) | 8.0 (7.6 - 8.5) | 7.3 (7.1 - 7.5) |

*F – Kidney cancer*

|  | **SACT database only SACT proportion, % (95% CI)** | **SACT database & HES SACT proportion, % (95% CI)** | **SACT database & HES & Cancer registry SACT proportion, % (95% CI)** | **NDRS SACT proportion, % (95% CI)** |
| --- | --- | --- | --- | --- |
| **2013** | 10.9 (9.0 - 12.8) | 14.0 (11.9 - 16.1) | 14.6 (12.4 - 16.7) | 13.4 (12.8 - 14.1) |
| **2014** | 10.8 (9.0 - 12.6) | 12.2 (10.3 - 14.1) | 13.8 (11.8 - 15.8) | 14.0 (13.4 - 14.7) |
| **2015** | 12.2 (10.4 - 14.1) | 14.4 (12.4 - 16.3) | 14.9 (12.9 - 17.0) | 14.3 (13.7 - 15.0) |
| **2016** | 11.1 (9.4 - 12.8) | 12.9 (11.1 - 14.8) | 13.3 (11.4 - 15.2) | 14.1 (13.5 - 14.8) |
| **2017** | 13.3 (11.5 - 15.1) | 14.2 (12.4 - 16.0) | 14.9 (13.0 - 16.7) | 15.1 (14.5 - 15.8) |
| **2018** | 12.3 (10.7 - 14.0) | 13.3 (11.6 - 15.1) | 13.9 (12.2 - 15.7) | 15.6 (14.9 - 16.3) |
| **2019** | 12.1 (10.5 - 13.7) | 13.4 (11.7 - 15.0) | 13.8 (12.1 - 15.5) | 15.5 (14.8 - 16.1) |
| **2020** | 13.1 (11.4 - 14.7) | 15.4 (13.6 - 17.1) | 15.7 (13.9 - 17.5) | 15.8 (15.2 - 16.5) |
| **Overall** | 12.1 (11.5 - 12.7) | 13.8 (13.1 - 14.4) | 14.4 (13.7 - 15.0) | 14.8 (14.5 - 15.0) |

*G – Pancreatic cancer*

|  | **SACT database only SACT proportion, % (95% CI)** | **SACT database & HES SACT proportion, % (95% CI)** | **SACT database & HES & Cancer registry SACT proportion, % (95% CI)** | **NDRS SACT proportion, % (95% CI)** |
| --- | --- | --- | --- | --- |
| **2013** | 23.2 (20.5 - 25.9) | 28.0 (25.1 - 30.9) | 29.0 (26.1 - 31.9) | 28.8 (27.8 - 29.7) |
| **2014** | 28.9 (26.1 - 31.7) | 31.4 (28.6 - 34.3) | 32.9 (30.0 - 35.8) | 28.2 (27.3 - 29.2) |
| **2015** | 24.3 (21.8 - 26.7) | 26.6 (24.1 - 29.2) | 27.2 (24.6 - 29.8) | 28.6 (27.7 - 29.6) |
| **2016** | 24.8 (22.3 - 27.3) | 26.5 (23.9 - 29.0) | 27.4 (24.8 - 30.0) | 28.1 (27.2 - 29.0) |
| **2017** | 27.7 (25.4 - 30.1) | 29.2 (26.8 - 31.6) | 30.6 (28.2 - 33.0) | 29.2 (28.3 - 30.1) |
| **2018** | 28.2 (25.8 - 30.6) | 29.8 (27.4 - 32.3) | 30.7 (28.2 - 33.1) | 29.5 (28.6 - 30.5) |
| **2019** | 26.8 (24.5 - 29.1) | 28.1 (25.8 - 30.4) | 28.7 (26.4 - 31.1) | 28.6 (27.7 - 29.5) |
| **2020** | 25.8 (23.6 - 28.0) | 27.3 (25.1 - 29.6) | 28.3 (26.1 - 30.6) | 27.4 (26.5 - 28.3) |
| **Overall** | 26.3 (25.4 - 27.2) | 28.3 (27.4 - 29.2) | 29.3 (28.4 - 30.2) | 28.5 (28.2 - 28.9) |

*H – Oesophageal cancer*

|  | **SACT database only SACT proportion, % (95% CI)** | **SACT database & HES SACT proportion, % (95% CI)** | **SACT database & HES & Cancer registry SACT proportion, % (95% CI)** | **NDRS SACT proportion, % (95% CI)** |
| --- | --- | --- | --- | --- |
| **2013** | 41.5 (38.4 - 44.6) | 48.2 (45.0 - 51.3) | 49.5 (46.4 - 52.7) | 44.0 (42.9 - 45.2) |
| **2014** | 44.5 (41.4 - 47.5) | 47.2 (44.1 - 50.2) | 48.9 (45.9 - 52.0) | 46.8 (45.6 - 47.9) |
| **2015** | 41.4 (38.5 - 44.3) | 44.5 (41.5 - 47.4) | 45.8 (42.9 - 48.8) | 45.8 (44.6 - 46.9) |
| **2016** | 47.4 (44.5 - 50.2) | 49.5 (46.7 - 52.4) | 50.8 (48.0 - 53.6) | 45.6 (44.5 - 46.7) |
| **2017** | 45.2 (42.4 - 47.9) | 46.8 (44.1 - 49.6) | 48.3 (45.5 - 51.1) | 46.1 (45.0 - 47.2) |
| **2018** | 45.7 (43.1 - 48.4) | 47.3 (44.6 - 50.0) | 48.8 (46.1 - 51.4) | 46.2 (45.2 - 47.3) |
| **2019** | 44.4 (41.8 - 47.0) | 45.3 (42.7 - 48.0) | 46.3 (43.7 - 49.0) | 45.1 (44.0 - 46.1) |
| **2020** | 44.7 (42.1 - 47.3) | 45.5 (42.9 - 48.1) | 46.8 (44.2 - 49.4) | 44.1 (43.0 - 45.2) |
| **Overall** | 44.5 (43.5 - 45.5) | 46.7 (45.7 - 47.7) | 48.1 (47.1 - 49.1) | 45.4 (45.1 - 45.8) |

*I – Gastric cancer*

|  | **SACT database only SACT proportion, % (95% CI)** | **SACT database & HES SACT proportion, % (95% CI)** | **SACT database & HES & Cancer registry SACT proportion, % (95% CI)** | **NDRS SACT proportion, % (95% CI)** |
| --- | --- | --- | --- | --- |
| **2013** | 32.0 (27.7 - 36.2) | 35.8 (31.5 - 40.2) | 37.3 (32.9 - 41.7) | 34.6 (33.4 - 35.8) |
| **2014** | 29.5 (25.4 - 33.6) | 32.3 (28.0 - 36.5) | 33.3 (29.1 - 37.6) | 35.7 (34.4 - 37.0) |
| **2015** | 29.3 (25.2 - 33.4) | 31.9 (27.7 - 36.1) | 32.5 (28.3 - 36.7) | 36.1 (34.9 - 37.4) |
| **2016** | 29.2 (25.2 - 33.1) | 30.8 (26.7 - 34.8) | 32.3 (28.3 - 36.4) | 35.0 (33.8 - 36.3) |
| **2017** | 32.2 (28.0 - 36.4) | 34.1 (29.9 - 38.3) | 36.0 (31.7 - 40.3) | 35.5 (34.2 - 36.8) |
| **2018** | 31.7 (27.7 - 35.6) | 32.6 (28.6 - 36.6) | 34.3 (30.3 - 38.3) | 33.3 (31.9 - 34.7) |
| **2019** | 27.9 (24.2 - 31.6) | 29.2 (25.4 - 32.9) | 29.7 (25.9 - 33.5) | 31.0 (29.6 - 32.3) |
| **2020** | 29.7 (25.8 - 33.6) | 31.2 (27.3 - 35.2) | 33.0 (28.9 - 37.0) | 29.8 (28.4 - 31.2) |
| **Overall** | 30.1 (28.7 - 31.6) | 32.1 (30.7 - 33.6) | 33.5 (32.0 - 34.9) | 34.1 (33.6 - 34.5) |

*Footnote. Incremental capture columns show cumulative coverage as sources are added - they are not mutually exclusive. The final step equals the QResearch-linked database analytic proportion with the NDRS national proportion presented for comparison.*

**Supplementary Figure 2**. Incremental contribution of data sources to radiotherapy (RT) ascertainment by cancer type and year (2013-2020).

*Footnote: Bars show cumulative radiotherapy ascertainment. Blue represents cases identified using HES procedure codes; purple represents additional cases identified through the cancer registry (NCRD) not captured in HES. The red line indicates the corresponding NDRS treatment estimate. Categories are mutually exclusive within each stack, with registry-derived cases representing those not identified in HES.*

*Abbreviations: HES - Hospital Episode Statistics.; NCRD – National Cancer Registration Dataset; NDRS – National Disease Registration Service; RT – radiotherapy.*

**Supplementary Table S4 (A-I)**. Radiotherapy (RT): QResearch-linked treated proportion and incremental source contributions by cancer type and year compared with NDRS data.

*A – Breast cancer*

|  | **HES database only RT proportion, % (95% CI)** | **HES & Cancer registry RT proportion, % (95% CI)** | **NDRS RT proportion, % (95% CI)** |
| --- | --- | --- | --- |
| **2013** | 40.9 (39.5 - 42.3) | 64.0 (62.7 - 65.4) | 63.1 (62.7 - 63.5) |
| **2014** | 42.8 (41.4 - 44.1) | 63.0 (61.6 - 64.3) | 62.8 (62.3 - 63.2) |
| **2015** | 42.4 (41.1 - 43.7) | 62.2 (60.9 - 63.5) | 62.5 (62.0 - 62.9) |
| **2016** | 42.6 (41.3 - 43.9) | 63.4 (62.1 - 64.7) | 63.1 (62.7 - 63.6) |
| **2017** | 44.4 (43.2 - 45.7) | 64.7 (63.5 - 65.9) | 63.1 (62.6 - 63.5) |
| **2018** | 41.6 (40.4 - 42.7) | 62.9 (61.8 - 64.1) | 61.7 (61.3 - 62.2) |
| **2019** | 39.6 (38.5 - 40.8) | 61.2 (60.1 - 62.4) | 60.1 (59.6 - 60.5) |
| **2020** | 37.4 (36.2 - 38.6) | 59.8 (58.5 - 61.0) | 58.4 (57.9 - 58.9) |
| **Overall** | 41.4 (41.0 – 41.9) | 62.6 (62.1 - 63.0) | 61.8 (61.7 - 61.9) |

*B – Prostate cancer*

|  | **HES database only RT proportion, % (95% CI)** | **HES & Cancer registry RT proportion, % (95% CI)** | **NDRS RT proportion, % (95% CI)** |
| --- | --- | --- | --- |
| **2013** | 22.9 (21.7 - 24.2) | 34.7 (33.3 - 36.1) | 31.9 (31.4 - 32.3) |
| **2014** | 23.3 (22.0 - 24.5) | 33.9 (32.5 - 35.3) | 31.3 (30.9 - 31.8) |
| **2015** | 24.2 (23.0 - 25.4) | 34.0 (32.7 - 35.3) | 31.6 (31.2 - 32.1) |
| **2016** | 22.6 (21.5 - 23.8) | 33.9 (32.6 - 35.2) | 32.4 (31.9 - 32.8) |
| **2017** | 22.0 (20.9 - 23.0) | 33.8 (32.5 - 35.0) | 31.5 (31.1 - 32.0) |
| **2018** | 22.9 (21.9 - 23.9) | 35.8 (34.7 - 37.0) | 34.1 (33.7 - 34.5) |
| **2019** | 21.5 (20.6 - 22.5) | 34.9 (33.7 - 36.0) | 32.4 (32.0 - 32.8) |
| **2020** | 21.7 (20.6 - 22.8) | 35.3 (34.1 - 36.6) | 33.0 (32.6 - 33.5) |
| **Overall** | 22.6 (22.2 – 23.0) | 34.6 (34.2 - 35.1) | 32.6 (32.5 - 32.7) |

*C – Colon cancer*

|  | **HES database only RT proportion, % (95% CI)** | **HES & Cancer registry RT proportion, % (95% CI)** | **NDRS RT proportion, % (95% CI)** |
| --- | --- | --- | --- |
| **2013** | 2.0 (1.5 - 2.6) | 4.7 (3.8 - 5.5) | 3.4 (3.2 - 3.6) |
| **2014** | 2.1 (1.6 - 2.7) | 5.0 (4.2 - 5.9) | 3.4 (3.2 - 3.7) |
| **2015** | 1.9 (1.4 - 2.4) | 3.8 (3.1 - 4.4) | 3.3 (3.0 - 3.5) |
| **2016** | 2.3 (1.7 - 2.8) | 4.4 (3.7 - 5.1) | 3.3 (3.0 - 3.5) |
| **2017** | 2.5 (2.0 - 3.1) | 4.0 (3.4 - 4.7) | 3.1 (2.9 - 3.3) |
| **2018** | 2.0 (1.6 - 2.5) | 4.0 (3.3 - 4.6) | 3.1 (2.9 - 3.3) |
| **2019** | 1.8 (1.4 - 2.2) | 3.6 (3.0 - 4.1) | 3.3 (3.1 - 3.6) |
| **2020** | 1.8 (1.4 - 2.2) | 3.2 (2.6 - 3.7) | 3.0 (2.8 - 3.3) |
| **Overall** | 2.0 (1.9 – 2.2) | 4.0 (3.8 - 4.2) | 3.1 (3.1 - 3.2) |

*D – Rectal cancer*

|  | **HES database only RT proportion, % (95% CI)** | **HES & Cancer registry RT proportion, % (95% CI)** | **NDRS RT proportion, % (95% CI)** |
| --- | --- | --- | --- |
| **2013** | 26.9 (24.2 - 29.6) | 40.1 (37.1 - 43.1) | 40.9 (39.9 - 41.9) |
| **2014** | 26.9 (24.2 - 29.6) | 38.1 (35.2 - 41.1) | 40.5 (39.5 - 41.5) |
| **2015** | 24.8 (22.2 - 27.4) | 33.4 (30.6 - 36.2) | 39.0 (38.0 - 39.9) |
| **2016** | 24.4 (22.0 - 26.9) | 37.7 (34.9 - 40.4) | 39.0 (38.0 - 40.0) |
| **2017** | 26.5 (24.0 - 28.9) | 40.6 (37.9 - 43.3) | 38.6 (37.6 - 39.6) |
| **2018** | 26.5 (24.1 - 28.9) | 39.9 (37.3 - 42.6) | 38.6 (37.7 - 39.6) |
| **2019** | 25.2 (23.0 - 27.4) | 38.4 (35.9 - 40.9) | 38.8 (37.8 - 39.7) |
| **2020** | 25.5 (23.2 - 27.9) | 39.6 (37.0 - 42.2) | 39.9 (38.9 - 40.9) |
| **Overall** | 25.8 (24.9 – 26.7) | 38.6 (37.6 - 39.5) | 39.4 (39.0 - 39.7) |

*E – Melanoma*

|  | **HES database only RT proportion, % (95% CI)** | **HES & Cancer registry RT proportion, % (95% CI)** | **NDRS RT proportion, % (95% CI)** |
| --- | --- | --- | --- |
| **2013** | 1.9 (1.2 - 2.6) | 3.5 (2.5 - 4.4) | 3.7 (3.4 - 4.0) |
| **2014** | 3.1 (2.3 - 4.0) | 4.4 (3.4 - 5.5) | 3.6 (3.3 - 3.9) |
| **2015** | 2.1 (1.4 - 2.8) | 3.3 (2.4 - 4.1) | 3.3 (3.0 - 3.6) |
| **2016** | 2.6 (1.8 - 3.3) | 3.9 (3.0 - 4.8) | 3.1 (2.8 - 3.4) |
| **2017** | 1.9 (1.3 - 2.5) | 3.1 (2.4 - 3.9) | 2.7 (2.5 - 3.0) |
| **2018** | 2.1 (1.5 - 2.7) | 2.8 (2.1 - 3.5) | 2.4 (2.1 - 2.6) |
| **2019** | 1.4 (0.9 - 1.9) | 2.0 (1.5 - 2.6) | 2.4 (2.2 - 2.7) |
| **2020** | 2.4 (1.7 - 3.1) | 3.1 (2.4 - 3.9) | 3.0 (2.7 - 3.3) |
| **Overall** | 2.1 (1.9 -2.4) | 3.2 (2.9 - 3.5) | 2.9 (2.8 - 2.9) |

*F – Kidney cancer*

|  | **HES database only RT proportion, % (95% CI)** | **HES & Cancer registry RT proportion, % (95% CI)** | **NDRS RT proportion, % (95% CI)** |
| --- | --- | --- | --- |
| **2013** | 4.5 (3.2 - 5.7) | 8.9 (7.1 - 10.6) | 7.9 (7.4 - 8.4) |
| **2014** | 5.5 (4.2 - 6.8) | 9.5 (7.9 - 11.2) | 7.5 (7.1 - 8.1) |
| **2015** | 4.6 (3.4 - 5.8) | 8.5 (6.9 - 10.1) | 7.4 (6.9 - 7.9) |
| **2016** | 5.9 (4.6 - 7.2) | 9.6 (8.0 - 11.2) | 7.6 (7.1 - 8.1) |
| **2017** | 4.8 (3.7 - 5.9) | 7.2 (5.9 - 8.6) | 7.1 (6.7 - 7.6) |
| **2018** | 3.5 (2.6 - 4.5) | 6.7 (5.4 - 8.0) | 6.8 (6.4 - 7.3) |
| **2019** | 4.6 (3.6 - 5.7) | 7.4 (6.2 - 8.7) | 6.4 (6.0 - 6.9) |
| **2020** | 4.7 (3.7 - 5.8) | 7.4 (6.1 - 8.7) | 7.3 (6.8 - 7.8) |
| **Overall** | 4.7 (4.3 – 5.1) | 8.0 (7.5 - 8.6) | 7.1 (6.9 - 7.2) |

*G – Pancreatic cancer*

|  | **HES database only RT proportion, % (95% CI)** | **HES & Cancer registry RT proportion, % (95% CI)** | **NDRS RT proportion, % (95% CI)** |
| --- | --- | --- | --- |
| **2013** | 2.3 (1.3 - 3.2) | 4.5 (3.2 - 5.8) | 5.5 (5.0 - 6.0) |
| **2014** | 3.8 (2.6 - 5.0) | 5.9 (4.4 - 7.3) | 4.7 (4.3 - 5.2) |
| **2015** | 2.6 (1.6 - 3.5) | 4.1 (2.9 - 5.2) | 5.0 (4.6 - 5.5) |
| **2016** | 3.8 (2.7 - 4.9) | 5.5 (4.1 - 6.8) | 5.2 (4.7 - 5.6) |
| **2017** | 2.7 (1.8 - 3.5) | 4.8 (3.6 - 5.9) | 5.1 (4.7 - 5.6) |
| **2018** | 2.9 (2.0 - 3.8) | 5.0 (3.8 - 6.2) | 5.8 (5.3 - 6.3) |
| **2019** | 3.3 (2.4 - 4.3) | 4.9 (3.8 - 6.1) | 5.2 (4.8 - 5.7) |
| **2020** | 4.0 (3.0 - 5.0) | 5.8 (4.6 - 6.9) | 5.7 (5.3 - 6.2) |
| **Overall** | 3.2 (2.9 – 3.5) | 5.1 (4.6 - 5.5) | 5.2 (5.1 - 5.4) |

*H – Oesophageal cancer*

|  | **HES database only RT proportion, % (95% CI)** | **HES & Cancer registry RT proportion, % (95% CI)** | **NDRS RT proportion, % (95% CI)** |
| --- | --- | --- | --- |
| **2013** | 16.3 (13.9 - 18.6) | 29.3 (26.4 - 32.2) | 30.5 (29.4 - 31.5) |
| **2014** | 17.7 (15.4 - 20.0) | 28.9 (26.1 - 31.7) | 30.4 (29.4 - 31.5) |
| **2015** | 17.9 (15.6 - 20.2) | 25.7 (23.1 - 28.3) | 30.7 (29.7 - 31.7) |
| **2016** | 19.5 (17.3 - 21.8) | 32.3 (29.6 - 35.0) | 31.8 (30.8 - 32.9) |
| **2017** | 17.9 (15.7 - 20.0) | 28.9 (26.4 - 31.4) | 30.2 (29.2 - 31.2) |
| **2018** | 16.0 (14.0 - 17.9) | 28.7 (26.2 - 31.1) | 29.4 (28.5 - 30.4) |
| **2019** | 17.4 (15.4 - 19.3) | 29.9 (27.5 - 32.3) | 29.3 (28.4 - 30.3) |
| **2020** | 18.0 (16.0 - 20.0) | 28.9 (26.6 - 31.3) | 29.7 (28.7 - 30.7) |
| **Overall** | 17.6 (16.8 – 18.3) | 29.1 (28.2 - 30.0) | 29.4 (29.1 - 29.7) |

*I – Gastric cancer*

|  | **HES database only RT proportion, % (95% CI)** | **HES & Cancer registry RT proportion, % (95% CI)** | **NDRS RT proportion, % (95% CI)** |
| --- | --- | --- | --- |
| **2013** | 4.3 (2.4 - 6.1) | 8.6 (6.0 - 11.1) | 9.6 (8.9 - 10.4) |
| **2014** | 2.5 (1.1 - 4.0) | 6.4 (4.2 - 8.6) | 11.3 (10.4 - 12.1) |
| **2015** | 3.4 (1.7 - 5.0) | 7.8 (5.4 - 10.2) | 10.9 (10.1 - 11.7) |
| **2016** | 4.2 (2.4 - 5.9) | 6.9 (4.7 - 9.2) | 10.6 (9.8 - 11.5) |
| **2017** | 3.7 (2.0 - 5.4) | 8.9 (6.4 - 11.5) | 9.9 (9.1 - 10.7) |
| **2018** | 4.3 (2.6 - 6.0) | 7.6 (5.4 - 9.8) | 8.3 (7.5 - 9.1) |
| **2019** | 3.4 (1.9 - 4.9) | 7.0 (4.9 - 9.1) | 7.3 (6.6 - 8.1) |
| **2020** | 4.2 (2.5 - 5.9) | 6.7 (4.5 - 8.8) | 7.7 (6.9 - 8.5) |
| **Overall** | 3.8 (3.2 – 4.3) | 7.5 (6.6 - 8.3) | 9.1 (8.9 - 9.4) |

*Footnote. Incremental capture columns show cumulative coverage as sources are added - they are not mutually exclusive. The final step equals the QResearch-linked database analytic proportion with the NDRS national proportion presented for comparison.*

**Supplementary Figure 3.** Distribution of cancer cases across linked data sources, England, 2013-2020. (**A**) Inclusive of all eligible cancer records diagnosed within study period, (**B**) sensitivity analysis limited to patients’ first cancer record only.

*Abbreviations: HES - Hospital Episode Statistics.*

**Supplementary Table S5**. Demographic and clinical characteristics of registry-only identified first cancers compared with all other linked cases, England, calendar years 2013-2020.

| **Demographics** | **Registry-only patients**  **(N%)** | **Non-registry only patients**  **N (%)** | **All eligible patients**  **N (%)** |
| --- | --- | --- | --- |
| **TOTAL CANCERS (row %)** | 7,550 (4.0) | 179,742 (96.0) | 187,292 |
| **CANCER TYPE** |  |  |  |
| Breast | 944 (12.5) | 50,219 (27.9) | 51,163 (27.3) |
| Prostate | 1,742 (23.1) | 49,221 (27.4) | 50,963 (27.2) |
| Colorectal | 1,739 (23.0) | 33,713 (18.8) | 35,452 (18.9) |
| Melanoma | 1,104 (14.6) | 15,001 (8.4) | 16,105 (8.6) |
| Kidney | 1,276 (16.9) | 9,907 (5.5) | 11,183 (6.0) |
| Pancreatic | 347 (4.6) | 9.283 (5.2) | 9,630 (5.1) |
| Gastro-oesophageal | 398 (5.3) | 12,398 (6.9) | 12,796 (6.8) |
| **SEX** |  |  |  |
| Male | 4,346 (57.6) | 94,979 (52.8) | 99,235 (53.0) |
| Female | 3,204 (42.4) | 84,763 (47.2) | 87,967 (47.0) |
| **AGE AT DIAGNOSIS (YEARS)** |  |  |  |
| <50 | 778 (10.3) | 17,274 (9.6) | 18,052 (9.6) |
| 50-59 | 939 (12.4) | 28,290 (15.7) | 29,229 (15.6) |
| 60-69 | 1,497 (19.8) | 46,308 (25.8) | 47,805 (25.5) |
| 70-79 | 2,137 (28.3) | 51,067 (28.4) | 53,204 (28.4) |
| 80+ | 2,199 (29.1) | 36,803 (20.5) | 39,002 (20.8) |
| **YEAR OF DIAGNOSIS** |  |  |  |
| 2013 | 672 (8.9) | 17,453 (9.7) | 18,125 (9.7) |
| 2014 | 753 (10.0) | 18,605 (10.4) | 19,358 (10.3) |
| 2015 | 753 (10.0) | 20,781 (11.6) | 21,534 (11.5) |
| 2016 | 904 (12.0) | 21,707 (12.1) | 22,611 (12.1) |
| 2017 | 926 (12.3) | 23,541 (13.1) | 24,467 (13.1) |
| 2018 | 988 (13.1) | 26,055 (14.5) | 27,043 (14.4) |
| 2019 | 1,260 (16.7) | 27,191 (15.1) | 28,451 (15.2) |
| 2020 | 1,294 (17.1) | 24,409 (13.6) | 25,703 (13.7) |
| **REGION** |  |  |  |
| East Midlands | 112 (1.5) | 3,503 (2.0) | 3,615 (1.9) |
| East of England | 283 (3.8) | 8,120 (4.5) | 8,403 (4.5) |
| London | 1,476 (19.6) | 30,564 (17.0) | 32,040 (17.1) |
| North East | 156 (2.1) | 5,050 (2.8) | 5,206 (2.8) |
| North West | 1,506 (20.0) | 39,211 (21.8) | 40,717 (21.7) |
| South East | 2,078 (27.5) | 48,385 (26.9) | 50,463 (26.9) |
| South West | 854 (11.3) | 18,745 (10.4) | 19,599 (10.5) |
| West Midlands | 886 (11.7) | 20,782 (11.6) | 21,668 (11.6) |
| Yorkshire & Humber | 199 (2.6) | 5,382 (3.0) | 5,581 (3.0) |
| **ETHNICITY** |  |  |  |
| White | 6,412 (91.5) | 153,554 (91.9) | 159,966 (91.9) |
| Asian | 253 (3.6) | 5,169 (3.1) | 5,422 (3.1) |
| Black | 189 (2.7) | 4,202 (2.5) | 4,391 (2.5) |
| Other | 154 (2.2) | 4,087 (2.5) | 4,241 (2.4) |
| Unknown | 542 | 12,730 | 13,272 |
| **STAGE** |  |  |  |
| Unstageable | 27 (0.5) | 158 (0.1) | 185 (0.1) |
| Stage 1 & 2 | 3,981 (75.3) | 82,319 (59.3) | 86,300 (59.9) |
| Stage 3 & 4 | 1,282 (24.2) | 56,367 (40.6) | 57,649 (40.0) |
| Unknown | 2,260 | 40,898 | 43,158 |
| **IMD QUINTILE** |  |  |  |
| 1 (most deprived) | 1,180 (15.6) | 24,628 (15.4) | 25,808 (15.4) |
| 2 | 1,440 (19.1) | 29,576 (18.5) | 31,016 (18.5) |
| 3 | 1,493 (19.8) | 32,303 (20.1) | 33,696 (20.1) |
| 4 | 1,612 (21.4) | 34,877 (21.8) | 36,489 (21.8) |
| 5 (least deprived) | 1,825 (24.2) | 38,907 (24.3) | 40,732 (24.3) |
| Unknown | 0 | 19,551 | 19,551 |
| **TREATED PROPORTIONS** |  |  |  |
| SACT-treated proportion (95% CI) | 5.3 (4.8 – 5.9) | 27.5 (27.3 – 27.7) | 26.7 (26.5 – 26.9) |
| RT-treated proportion (95% CI) | 7.0 (6.3 – 7.6) | 32.8 (32.5 – 33.0) | 31.8 (31.6 – 32.1) |

*Footnote: Data represents first cancer records present in the Cancer Registry only (“registry-only”) versus those also identified in QResearch, HES or death records. Percentages calculated within known categories to allow for comparison. P-values not shown as comparison is descriptive. Values represent counts (%) unless otherwise indicated. Column percentages reported unless otherwise specified.*

*Abbreviations: IMD – Index of Multiple Deprivation; RT – radiotherapy; SACT – systemic anti-cancer therapy; 95% CI – 95% confidence interval*
